# Metabolite Profiling and Transcriptome Analysis Explains Difference in Accumulation of Bioactive Constituents in Licorice (*Glycyrrhiza uralensis*) Under Salt Stress

**DOI:** 10.3389/fpls.2021.727882

**Published:** 2021-10-07

**Authors:** Chengcheng Wang, Lihong Chen, Zhichen Cai, Cuihua Chen, Zixiu Liu, Shengjin Liu, Lisi Zou, Mengxia Tan, Jiali Chen, Xunhong Liu, Yuqi Mei, Lifang Wei, Juan Liang, Jine Chen

**Affiliations:** ^1^School of Pharmacy, Nanjing University of Chinese Medicine, Nanjing, China; ^2^School of Pharmacy, Jiangsu Vocational College of Medicine, Yancheng, China

**Keywords:** licorice, salt stress, bioactive constituents, transcriptomics, biosynthesis network

## Abstract

Salinity stress significantly affects the contents of bioactive constituents in licorice *Glycyrrhiza uralensis*. To elucidate the molecular mechanism underlying the difference in the accumulation of these constituents under sodium chloride (NaCl, salt) stress, licorice seedlings were treated with NaCl and then subjected to an integrated transcriptomic and metabolite profiling analysis. The transcriptomic analysis results identified 3,664 differentially expressed genes (DEGs) including transcription factor family MYB and basic helix-loop-helix (*bHLH*). Most DEGs were involved in flavonoid and terpenoid biosynthesis pathways. In addition, 121 compounds including a triterpenoid and five classes of flavonoids (isoflavone, flavone, flavanone, isoflavan, and chalcone) were identified, and their relative levels were compared between the stressed and control groups using data from the ultrafast liquid chromatography (UFLC)–triple quadrupole–time of flight–tandem mass spectrometry (TOF–MS/MS) analysis. Putative biosynthesis networks of the flavonoids and triterpenoids were created and combined with structural DEGs such as phenylalanine ammonia-lyase (*PAL*), 4-coumarate-CoA ligase [*4CL*], cinnamate 4-hydroxylase [*C4H*], chalcone synthase [*CHS*], chalcone-flavanone isomerase [*CHI*], and flavonoid-3′,5′ hydroxylase (*F3*′*,5*′*H*) for flavonoids, and *CYP88D6* and *CYP72A154* for glycyrrhizin biosynthesis. Notably, significant upregulation of UDP-glycosyltransferase genes (*UGT*) in salt-stressed licorice indicated that postmodification of glycosyltransferase may participate in downstream biosynthesis of flavonoid glycosides and triterpenoid saponins. Accordingly, the expression trend of the DEGs is positively correlated with the accumulation of glycosides. Our study findings indicate that key DEGs and crucial *UGT* genes co-regulate flavonoid and saponin biosynthesis in licorice under salt stress.

## Introduction

Licorice (*Glycyrrhiza uralensis*) is considered to be one of the most economically important medicinal plants worldwide. There is a large amount of numerous bioactive constituents that have been obtained from species of the genus *Glycyrrhiza*, and they mainly include triterpenoids, flavonoids, and coumarin with pharmacological properties including detoxifying, anti-inflammatory, and hepatoprotective (Wang et al., 2015). In addition to clinical prescriptions, glycyrrhizin, a sweet and unique saponin extracted from licorice, and its derivatives are used as food additives and for several different applications (Nassiri-Asl and Hosseinzadeh, 2012). Although there are different ways of using licorice, high-value bioactive constituents of licorice primarily contribute to its medicinal benefits and commercial usefulness (Song et al., 2017). Approximately 11% of the bioactive constituents in licorice are saponins and flavonoids with diverse structures accounting for > 50%, including flavonols, isoflavones, flavanones, isoflavanones, isoflavans, chalcone, and their corresponding glycosides (Wang C. et al., 2020).

Previous studies have provided valuable proteomic information for elucidating the molecular mechanism underlying flavonoid and triterpenoid metabolism under salt-stress conditions (Yu et al., 2015; Wang C. C. et al., 2020). However, research on key genes regulating the biosynthesis of these constituents in licorice growing naturally under high salinity conditions still remains limited. Presently, the whole-genome sequence of *G. uralensis* has been published and potential genomic information about molecules involved in the biosynthesis of bioactive constituents has been identified (Mochida et al., 2017). These findings provide necessary and important genetic clues for developing strategies for effective molecular breeding and crop-quality control (Mochida et al., 2017). Currently, several key enzymes including cytochrome P450 88D6 (CYP88D6), CYP72A154, UDP-glycosyltransferase (UGT73P12), and GuCSyGT have been successfully characterized in the downstream biosynthetic pathway of glycyrrhizin (Seki et al., 2008, 2011; Chung et al., 2020). A comprehensive understanding of the regulatory mechanisms involved in the synthesis of bioactive constituents in licorice is critical to the breeding of high-quality cultivated licorice. Hence, it is extremely important to characterize the key genes in the biosynthetic pathways of these constituents of licorice.

In the present study, we investigated the molecular mechanisms involved in the biosynthesis of the chemical components of licorice cultivated under salt-stress conditions with a focus on qualitative differences, using an integrative transcriptomic analysis and metabolomic approach. Differentially expressed genes (DEGs) among the structural genes involved in flavonoid and triterpenoid saponin biosynthesis were identified using bioinformatics analysis and putative biosynthesis networks were created. Two crucial *UGT* genes, *Glyur000289s00018722* and *Glyur000121s00009707* were identified as the most promising DEGs for further regulatory network study. This work will not only facilitate the elucidation of flavonoid and triterpenoid saponin biosynthesis under high salinity stress conditions at both the metabolic and gene levels, but it will also contribute to the further development of high-quality cultivated licorice.

## Materials and Methods

### Plant Materials and Salinity Treatments

The salinity treatments used were those referenced in our previous study (Wang C. C. et al., 2020). Briefly, *G. uralensis* Fisch cultivars (1-year-old) were collected from Yanchi County, Ningxia Province, China. We chose licorice seedlings with a similar diameter and a number of buds, which were planted in the Medicinal Botanical Garden of Nanjing University of Traditional Chinese Medicine (northern latitude 118°57′1″, east longitude 32°6′5″) under a transparent film shelter, protected from rainwater. Two licorice seedlings were planted in each pot (top and bottom diameter, 30 and 25 cm, respectively, height 50 cm) with approximately 25 kg dry soil [texture, loam; organic carbon, 36.6 g/kg; cation exchange capacity, 17.0 cmol (+)/kg^–1^; pH, 5.0] (Chen et al., 2018).

The experimental licorice plants were allowed to grow until their buds broke. Then, two salt concentration levels, 0 mM (control group) and 50 mM NaCl (treatment group) were designed with four replicates (two seedlings in each pot) at each concentration level. Salt concentrations were increased gradually until the designated concentration was reached, and the whole salt-stress process lasted for 50 days (Supplementary Figure 1). Finally, experimental licorice plant samples were harvested, the same parts of the root tissues were collected, and then they were cleaned with phosphate-buffered saline (PBS). Two parallel samples were prepared, one was cut with sterilized scissors and quickly frozen in liquid nitrogen for RNA sequencing (RNAseq) and the other was naturally dried for metabolite determination. The remaining plants were collected and deposited as voucher specimens (Zhao et al., 2018; Wang C. C. et al., 2020).

### Metabolite Profiling Analysis of Bioactive Constituents in Licorice

The sample preparation process used was a modification of a previous experimental method we used (Wang et al., 2019). Briefly, all samples were powdered by crushing and then screened through a 60-mesh sieve. Each sample was accurately weighed (0.5 g) and subsequently extracted with 25 mL 70% methanol for 1 h using ultrasonication (500 W, 40 kHz). After cooling the extract at room temperature for 15 min, methanol was added to make up for the weight loss during extraction. The mixture was then centrifuged at 12,000 rpm for 10 min and the supernatant was collected, diluted 10-fold with the solvent, and filtered using a 0.22 μm membrane filter prior to performing the ultrafast liquid chromatography (UFLC)–triple time-of-flight tandem mass spectrometry (TOF-MS/MS) analysis.

All samples were analyzed using a UFLC system (Shimadzu, Kyoto, Japan) interfaced with a triple TOF–MS system equipped with an electrospray ionization (ESI) source. An Agilent ZORBAX Extend-C18 column (100 mm × 2.1 mm, 1.8 μm) was used for all the analyses. The mobile phase was composed of a 0.1% formic acid aqueous solution (A) and acetonitrile (B) and was run at a flow rate of 0.3 mL/min with the column temperature maintained at 35°C. Gradient elution was optimized and performed according to the following schedule: 10–35% B at 0–23 min, 35–45% B at 23–26 min, 45–50% B at 26–29 min, 50–55% B at 29–32 min, 55–65% B at 32–36 min, 65–75% B at 36–40 min, 75–95% B at 40–45 min, and 2% B at 45–48 min. The injection volume of the reference compounds and samples was 2 μL (Wang et al., 2019).

The Triple TOF^TM^ 5600-MS/MS system (AB Sciex, Framingham, MA, United States), which was equipped with an ESI source, was used to acquire the MS data. The positive ion mode was adopted with a capillary voltage of 5.0 kV, and the scanning range of each sample was from *m*/*z* 50 to 1,500. The optimized MS parameters were as follows: flow rate of curtain gas was 40 L/min, ion source temperature was 550°C, the flow rate of nebulization gas was 55 L/min, the flow rate of auxiliary gas was 55 L/min, the spray voltage was −4,500 V, and the declustering potential voltage was 100 V (Wang et al., 2019).

The LC-triple TOF-MS/MS data were collected in the negative ion mode using the Analyst TF 1.6 software and analyzed using PeakView1.2 software (both from AB Sciex) to identify the potential chemicals in the control and salt-stressed licorice samples. The analysis of the multistage MS/MS allowed identification of the characteristic peaks with MS data singular point excluding, noise filtering, peak matching, peak recognition, baseline correction, peak alignment, and extraction of the characteristic peak. After matching the compounds according to the information in the established database, they were identified. Ions with the same *m/z* value in different samples were set at a maximum tolerance of 10 ppm and the same retention time (tR) was set at a tolerance of 0.2 min (Wang et al., 2019). Compounds were quantified based on means of the relative peak area and all analyses were conducted in triplicates (Rizzato et al., 2017; Zhao et al., 2018).

### RNA Isolation and cDNA Library Construction

RNA-seq was performed on three individual biological replicates from each group. Briefly, total RNA from the frozen licorice root was extracted using the Trizoltotal RNA isolation kit (Sangon Biotech, Shanghai, China) according to the protocols (Li H. et al., 2019). The concentration and purity of the extracted total RNA were detected using a Nanodrop 2000 spectrophotometer (Nanodrop Technologies, Thermo Fisher, United States). The RNA integrity was detected using agarose gel electrophoresis, and the RNA integrity number was determined using the Agilent 2100 bioanalyzer system (Agilent Technologies, CA, United States).

A cDNA library for sequencing requires 1 μg total RNA, a concentration of ≥ 50 ng/μl, and a value of the ratio of the optical density at a wavelength of 260–20 nm (OD260/280) between 1.8 and 2.2. A–T base pairing with Oligo (dT) magnetic beads and ployA (Invitrogen, CA, United States) was used to isolate mRNA from total RNA for the analysis of the transcriptome information. Purified mRNA was cleaved into small fragments and the first-strand cDNAs were synthesized using reverse transcription and random primers, followed by double-strand cDNAs synthesis. Then, the double-stranded cDNAs were purified, end-repaired, ligated to adaptors, further fragmented (Li H. et al., 2019), and then the cDNA fragments were finally amplified using polymerase chain reaction (PCR) to construct the cDNA library for the Illumina sequencing (Liu et al., 2020).

### Transcriptome Sequencing and Functional Annotation

The raw data were first filtered to remove low-quality sequences and contaminated adaptors. Then, the high-quality data were mapped to the reference genome sequence of *G. uralensis*^1^ using HISAT2 software (version 2.1.0) with default parameter settings. Furthermore, new genes/transcripts were obtained by reassembling the sequences and then comparing them with those in the public database, and then Cufflinks^2^ were used to acquire the unigenes. All assembled unigenes were aligned against the non-redundant National Center for Biotechnology Information (NCBI) protein database (Nr, version 2019) (Zhang et al., 2019), Swiss-Prot (version 2019), Pfam (version v32.0), and clusters of orthologous groups (COGs, version 5.0) using the basic local alignment search tool (BLASTX). Blast2go (version 2.5) and KOBAS (version 3.0) were used for Gene Ontology (GO, ^3^) function annotation, and Kyoto Encyclopedia of Genes and Genomes (KEGG, ^4^) was used to identify related metabolic pathways.

### Differentially Expressed Gene Analysis

The fragments per kilobases per million reads (FPKM) method was performed using the RSEM software (^5^, version 1.3.1) for differential expression analysis through estimation of the expression level. Furthermore, a |log2(fold change) ≥ 1 calculated using FPKM values was used as the threshold for significant differences in unigenes expression. False discovery rate (FDR) was to determine the *p*-value with the significance level set to < 0.05 after multiple testing. GO enrichment analysis was performed to distribute the DEGs into the following three categories: molecular function (MF), biological process (BP), and cellular component (CC). Moreover, KEGG enrichment analysis was used to identify the DEGs involved in major KEGG pathways (Liu et al., 2020; Ma et al., 2020). To explore the different accumulation mechanisms of the bioactive metabolites, candidate DEGs encoding enzymes associated with terpenoid and flavonoid biosynthesis were also identified based on the enriched KEGG pathways and gene functional annotation.

### Real-Time Quantitative Reverse Transcription PCR

We used real-time quantitative reverse transcription PCR (qRT-PCR) to quantify the relative mRNA expression patterns of the 11 genes (phenylalanine ammonia-lyase [*PAL*], *4CL*, chalcone synthase[*CHS*], chalcone–flavanone isomerase [*CHI*], *CYP93C*, *HIDH*, *HI4OMT*, *CYP72A154*, *CYP88D6*, and *bAS*), whose corresponding proteins are key enzymes encoding glycyrrhizin and flavonoid biosynthesis. Total RNA was extracted using the Trizol total RNA isolation kit. The primers used were designed using Primer 3.0 software^6^. cDNA pools from the total RNA were synthesized for the qRT-PCR using HiScript Q RT SuperMix for qPCR (Vazyme Biotech Co., Ltd., Nanjing, China). The 20.1 μL reaction volume contained 16.5 μL 2 × ChamQ SYBR color qPCR Master Mix, 0.8 μL of each primer (5 μM), and 2 μL cDNA. The PCR procedure was scheduled as follows: 95°C for 5 min, then 40 cycles at 95°C for 5 s, 60°C for 30 s, and 72°C for 40 s. β-Actin was used as the reference standard and the relative gene expression level was calculated using a real-time PCR system (ABI 7500 real-time PCR system) using the 2^–ΔΔCt^ method. Primers for the qRT-PCR are shown in Supplementary Table 1.

## Results

### Ultrafast Liquid Chromatography-Triple Time-of-Flight Tandem Mass Spectrometry Analysis of Bioactive Constituents in Licorice

To investigate differences in the expression patterns of multiple constituents between salt-stressed and untreated licorice, we analyzed crude licorice extracts from each group using UFLC-triple TOF-MS/MS based on our previously established method. Representative total ion chromatograms of the two groups are shown in Supplementary Figure 2. The UFLC-triple TOF-MS/MS data were collected using the Analyst TF 1.6 software (AB Sciex) (Wang et al., 2019). We identified 121 putative constituents using PeakView1.2 software (AB Sciex) according to their tRs, accurate mass, and MS/MS fragmentation characteristics in combination with a local home-built library search and comparison with standards and related literature reports (Supplementary Table 2).

**FIGURE 1:**
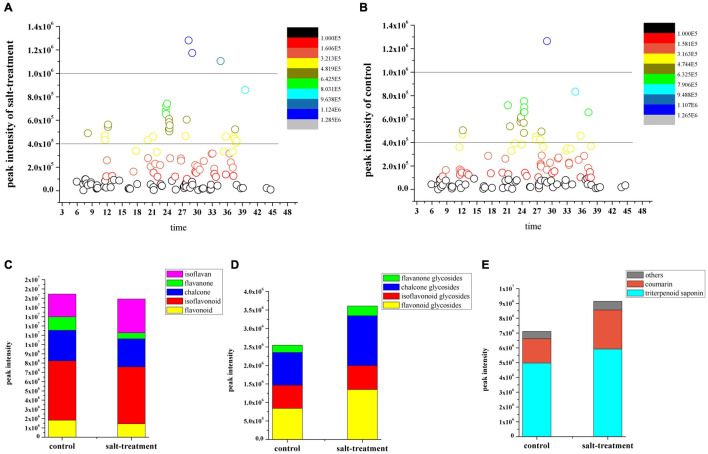
Distribution of bioactive constituents identified using ultrafast liquid chromatography (UFLC)-triple time-of-flight tandem mass spectrometry in **(A)** salt-stressed group and **(B)** control group based on their retention time. Total content changes in **(C)** flavonoid, **(D)** flavonoid glycoside, **(E)** triterpenoid saponin, coumarin, and other constituents between two groups.

The result showed differences between the metabolite profiles of multiple constituents of the salt-stressed licorice and those of the control samples (Figures 1A,B). The relative contents of flavonoids, triterpenoid saponins, glycosides, coumarin, and other constituents were obtained by calculating the means of the relative peak area of three replicates (Supplementary Table 3). The constituents were also accumulated differently in each group. Specifically, the content of glycosides displayed an opposite result from that of the corresponding aglycons, whereas flavonoid aglycons were present at relatively high levels in the licorice without salt treatment (Figures 1C,D and Supplementary Figure 4).

In addition, the levels of triterpenoid saponins, coumarin, and several other chemicals were found to be higher in salt-treated than control licorice samples (Figure 1E and Supplementary Figure 4). The analysis of the relative levels of multiple constituents in the licorice samples indicated that the contents of most identified metabolites obviously changed during salinity stress. Therefore, detailed information on the metabolite profile of licorice could be analyzed and further integrated with transcriptomic data to demonstrate its quality.

### Transcriptome Data and Global Analysis of Differentially Expressed Genes

As shown in Supplementary Table 4, raw and clean reads ranging from 48.7 to 58.3 million and 48.2 to 57.6 million, respectively, were obtained for each library. The Q30 percentage (sequences with sequencing error rate < 0.1%) (Li H. et al., 2019) was > 95% for each library, and the average GC content was approximately 46% for all libraries. Furthermore, 88.64–91.99% of the clean reads were mapped to the reference genome for each library. Overall, the results suggested that the RNA-seq data was high-quality and could be used for further analysis.

**FIGURE 2:**
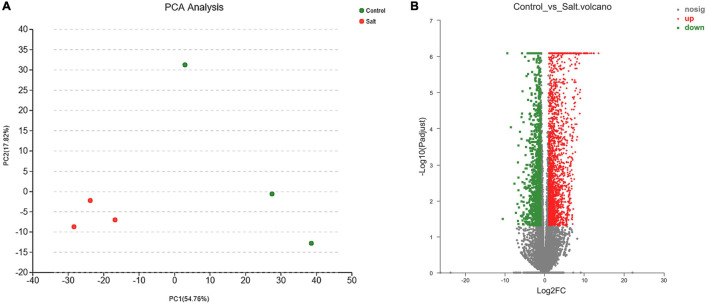
**(A)** Principal component analysis (PCA) of salt-stressed and control groups. **(B)** Volcano map analysis of differentially expressed genes (red, green, and gray points represent upregulated, downregulated, and unchanged genes, respectively).

The PCA (Figure 2A and Supplementary Figure 3) showed that the two groups of samples could be well separated and three replicates were gathered together by clustering, indicating that the samples had good repeatability, and the correlation among samples in each group was desirable. The pairwise comparison with a |log_2_(fold change)| ≥ 1 and P-adjusted < 0.05 as the threshold (Li H. et al., 2019), identified 3,664 DEGs consisting of 2,401 upregulated and 1,263 downregulated in the control vs. salt-treated groups (Figure 2B and Supplementary Table 5).

### Gene Ontology and Kyoto Encyclopedia of Genes and Genomes Enrichment Analysis of Differentially Expressed Genes

**FIGURE 3:**
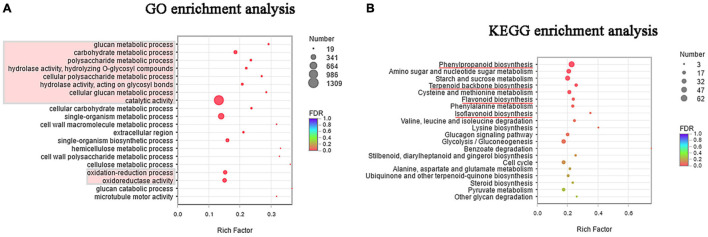
**(A)** Gene ontology (GO) enrichment of top 20 terms between salt-stressed and control groups. **(B)** Kyoto Encyclopedia of Genes and Genomes (KEGG) enrichment of top 20 pathways between salt-stressed and control groups.

To understand the biological function of the DEGs, GO term enrichment was performed using BLAST-GO (Li H. et al., 2019). The top 20 functional groups are shown in Figure 3, where the most genes enriched were those related to catalytic activity, and those related to metabolic processes of glucose also constituted a great proportion. In the cellular component category, cell part, cell, and organelle were the most highly represented terms. Moreover, Figure 3A shows that many genes under the oxidation-reduction process and oxidoreductase activity were enriched. This observation indicates that salinity may cause cellular ion toxicity, and the salt-stressed licorice likely developed an efficient defensive strategy by producing a series of enzymes to minimize the risk of oxidative stress (Wang C. C. et al., 2020).

Likewise, Figure 3B illustrates the top 20 categories enriched in the KEGG pathways. Apart from starch and sucrose metabolism pathways (ko00500, 43 genes), the other pathways were primarily related to the metabolism of amino acids and sugars, including amino sugar and nucleotide sugar metabolism (ko00520); cysteine and methionine metabolism (ko00270); phenylalanine metabolism (ko00360); valine, leucine, and isoleucine degradation (ko00280); lysine biosynthesis (ko00300); alanine, aspartate, and glutamate metabolism (ko00250); and degradation of other glycans (ko00511). Notably, pathways in the biosynthesis of important secondary metabolites were also significantly enriched and the top three were phenylpropanoid biosynthesis (ko00940, 62 genes), terpenoid backbone biosynthesis (ko00900, 20 genes), and flavonoid biosynthesis (ko00941, 18 genes). In addition, isoflavonoid biosynthesis was also identified in enriched pathways with a high rich factor, although this term was not statistically significant (*P*-corrected = 0.064).

### Analysis of Differentially Expressed Genes and Metabolites Involved in the Flavonoid Synthesis Pathway

**FIGURE 4:**
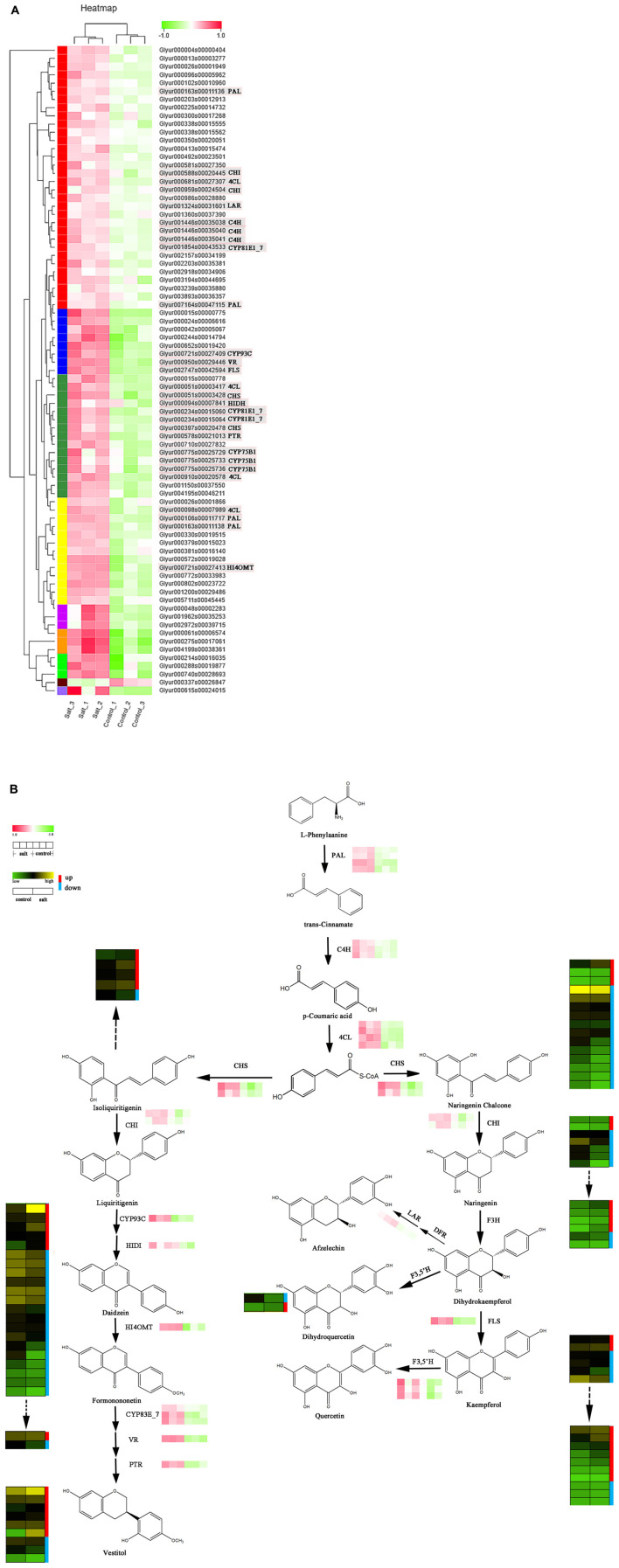
**(A)** Expression levels of structural differentially expressed genes (DEGs) involved in flavonoid biosynthesis. **(B)** Correlation analysis of expression pattern of key DEGs and metabolite profiles of flavonoids in licorice.

Differentially expressed genes that encode enzymes involved in the (iso)flavonoid biosynthesis pathway were identified based on the enriched KEGG pathways and gene functional annotation (Supplementary Table 6). The result showed that 79 structural DEGs were remarkably upregulated in the control vs. the salt-stressed group except for one gene (E1.11.1.7). Among the identified enzymes, genes of the following crucial rate-limiting enzymes were selected for further gene-chemical study: four putative *PAL*, four *4CL*, three cinnamate 4-hydroxylase*s* [*C4H*], three flavonoid-3′ hydroxylases [*F3*′*H*], three *CYP81E1_7*, two *CHS*, two *CHI*, and one *CYP93C*, *LAR*, *HIDH*, *HI4OMT*, flavone synthase [*FLS*], *VR*, and *PTR* (Figure 4A).

Figure 4 displays a putative network of (iso)flavonoid biosynthesis, which also shows the downstream flavanone and various glycosides. In the present study, four putative PALs were obtained, i.e., Glyur000163s00011136, Glyur007164s00047115, Glyur000106s00011717, and Glyur000163s00011138, and all four unigenes were significantly upregulated after salt stress.

The CH4 enzyme of the phenylpropanoid pathway is an important member of the CYP73 family that catalyzes *trans-*cinnamic acid hydroxylation into p-coumaric acid, ultimately providing crude material for the synthesis of diverse phenolic metabolites (Xu et al., 2010; Zhang et al., 2019). Subsequently, 4CL participates in the biosynthesis of flavonoids in the downstream steps of the pathway, converting p-coumaric acid into p-coumaroyl CoA, which is a substrate for the synthesis of various flavonoid constituents (Kumar et al., 2013). Three putative C4Hs (Glyur001446s00035038, Glyur001446s00035040, and Glyur001446s00035041) and four 4CLs (Glyur000681s00027307, Glyur000051s00003417, Glyur000910s00020578 and Glyur000098s00007989) were identified in our study. Similarly, these enzymes all exhibited higher expression levels in salt-stressed licorice than they did in the control samples.

The DEG analysis of flavonoid biosynthesis also showed an obvious difference between the expression profiles of the salt-treated and control groups. CHS, the first rate-limiting enzyme in the flavonoid biosynthesis pathway, mediates the isomerization of naringenin chalcone or isoliquiritigenin by catalyzing the condensation of p-coumaroyl-CoA and malonyl-CoA. In the present study, the two DEGs mapped to CHS were Glyur000051s00003428 and Glyur000397s00020478, and the expression levels of both were markedly increased by more than twofold (Supplementary Table 6). After the identification of CHS, two putative CHIs, Glyur000959s00024504 and Glyur005711s00045445, were obtained and shared by naringenin chalcone and isoliquiritigenin. They showed similar expression profiles to those of the CHSs. However, the chemical products of chalcone and flavanone were downregulated and not in accordance with the expression of corresponding genes such as *CHS*, *CHI*, and the corresponding upstream DEGs (Figure 4B and Supplementary Figure 4).

The flavonoid biosynthesis pathway then diverges into side branches producing two leading products (naringenin chalcone and isoliquiritigenin), and isoliquiritigenin is the precursor of the isoflavonoid class of compounds. Putative *CYP93C* (Glyur000721s00027409) and *HIDH* (Glyur000094s00007841) genes can convert liquiritigenin into daidzein, which is the first compound in the production of other isoflavonoids (Yuk et al., 2016). The next putative DEG is *HI4OMT* (Glyur000721s00027413), which adds a -CH3 group to the B-ring to yield formononetin (Yu et al., 2015). At this level, the chemical analysis suggested that most isoflavonoids showed lower relative contents in the salt-treated group than they did in the control group, which was also found to be in contrast to the results of the analysis of the associated DEGs.

Following isoflavonoid biosynthesis, isoflavan can be obtained from formononetin through several catalyzing steps regulated by *CYP81E1_7*, *VR*, and *PTR* genes. Three putative *CYP81E1_7* genes, *Glyur001854s00043533*, *Glyur000234s00015060*, and *Glyur000234s00015064*; one putative *VR* (*Glyur000950s00029446*); and one putative *PTR* (*Glyur000578s00021013*) were identified as DEGs and they showed similar expression patterns in two groups of licorice. The variations in isoflavan constituents were consistent with changes in the DEGs.

In the other branch of flavonoid biosynthesis, a putative FLS (Glyur002747s00042594) contributes to converting dihydroflavonols to kaempferol (Figure 4B; Nabavi et al., 2020) showed enhanced expression levels after salt stress. This finding indicates that the expression level of FLS was also not correlated with the variation in the flavone content. Furthermore, F3′Hs catalyze hydroxylation at the 3′ position of the B-ring of flavonoids (Wei et al., 2015; Zhang et al., 2019). As shown in Figure 4A, all three F3′Hs, Glyur000775s00025729, Glyur000775s00025733, and Glyur000775s00025736, exhibited higher expression levels in the salt-stressed licorice than in the control licorice.

### Analysis of Differentially Expressed Genes and Metabolites Involved in the Terpenoid Synthesis Pathway

**FIGURE 5:**
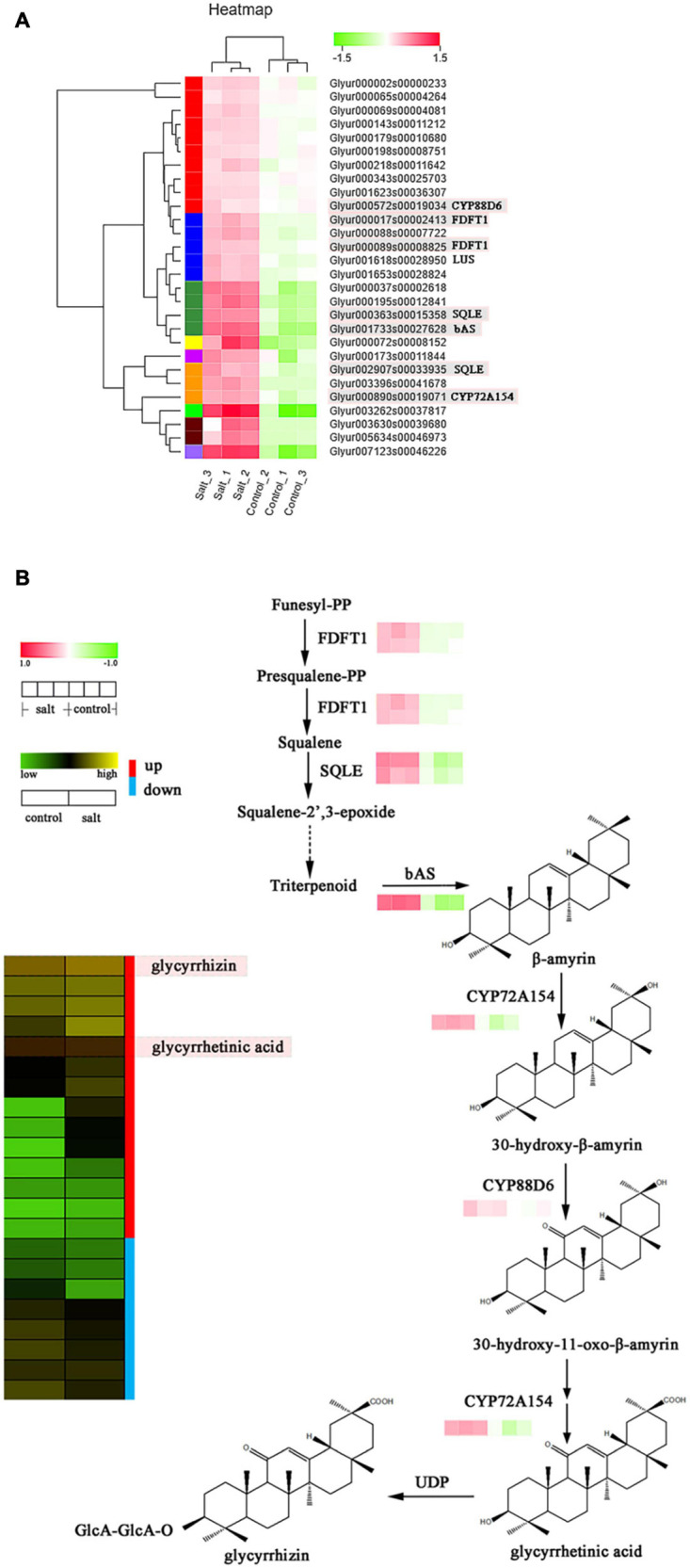
**(A)** Expression levels of structural differentially expressed genes (DEG) involved in triterpenoid saponin biosynthesis. **(B)** Correlation analysis of expression patterns of key DEGs and metabolite profiles of triterpenoid saponins in licorice.

In our study, we explored the differences in the accumulation of terpenoid metabolites by screening candidate enzyme genes involved in terpenoid synthesis or modification using DEGs. The findings suggested that 28 genes showed the same enhanced expression patterns after salt stress (Figure 5A), and terpenoid backbone biosynthesis was the primary pathway involved in the activity of the most upregulated synthetase genes (Supplementary Table 7). Furthermore, *bAS*, *CYP88D6*, and *CYP72A154* were the most important functional genes associated with the backbone synthesis, C-11, and C-30 oxidative reactions of glycyrrhizin, respectively. These genes were all dramatically upregulated (log_2_FC > 2 for CYP72A154, log_2_FC > 3 for CYP88D6, and log_2_FC > 5 for bAS) by NaCl treatment. Furthermore, 3-hydroxy-3-methylglutaryl coenzyme A reductase (EC 1.1.1.34, *HMGR*), 4-hydroxy-3-methylbut-2-en-1-yl diphosphate reductase (EC:1.17.7.4, ispH), *GGPS*, and other key genes mapped to the terpenoid backbone biosynthesis were upregulated under salt-stress conditions, indicating that these genes might influence the supply of precursors to regulate the biosynthesis of triterpenoids in licorice.

Triterpenoid saponins are another important class of licorice products with a wide range of biological activities (Thimmappa et al., 2014). The biosynthesis of diverse triterpenoids in licorice involves the activity of CYP 450 enzymes in oxidative reactions (Ramilowski et al., 2013; Renault et al., 2014; Wang C. C. et al., 2020). In this study, derivatives of glycyrrhizin in its synthetic pathway were identified as precursor compounds of most other triterpenoid saponins. Thus, a putative gene-chemical correlation network of glycyrrhizin biosynthesis was created, which consisted of seven significantly upregulated candidate genes (Figure 5). Two key CYP450 genes, *CYP88D6* (*Glyur001733s00027628*) and *CYP72A154* (*Glyur000890s00019071*) and a putative *bAS* (*Glyur000572s00019034*) were remarkably upregulated in the salt-treated group. Moreover, glycyrrhetinic acid and glycyrrhizin showed higher expression levels in the same group (Figure 5 and Supplementary Figure 4). In addition, 20 putative terpene synthase candidates involved in terpenoid backbone biosynthesis (such as HMGR, ispH, GGPS, and ispS, Supplementary Table 7) by catalyzing the synthesis of triterpene precursors, were also upregulated and may contribute to the biosynthesis of glycyrrhizin and other triterpenoids (Uthup et al., 2013). Most of the terpenoids, detected by analyzing the UFLC-MS/MS data, showed similar upregulated expression levels compared to those of the structural DEGs (Figure 5 and Supplementary Figure 4).

### Confirmation of Differentially Expressed Genes of Flavonoid and Triterpenoid Biosynthesis Using Real-Time Quantitative Reverse Transcription PCR

**FIGURE 6:**
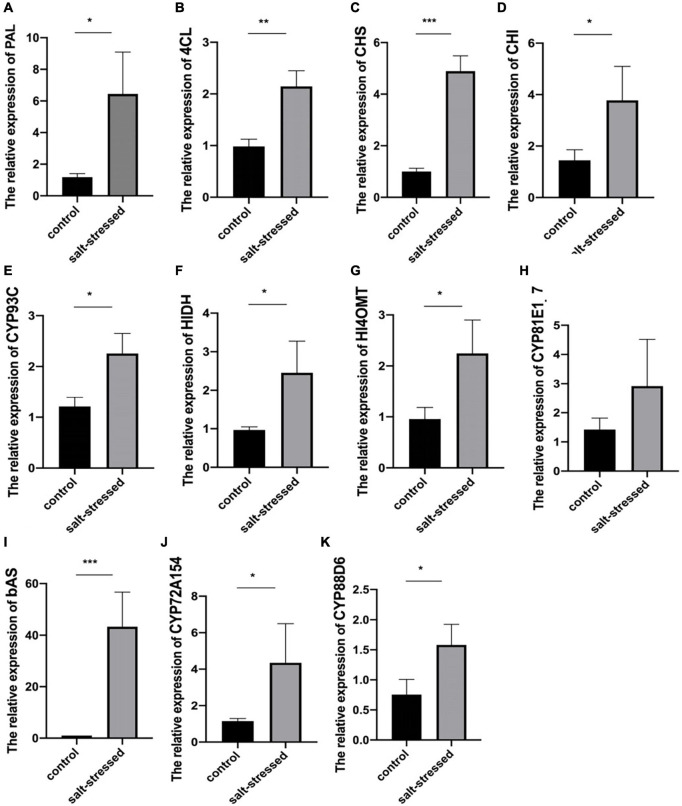
Difference in the expression levels of **(A)** PAL, **(B)** 4CL, **(C)** CHS, **(D)** CHI, **(E)** CYP93C, **(F)** HIDH, **(G)** HI4OMT, **(H)** CYP81E1_7, **(I)** bAS, **(J)** CYP72A154, and **(K)** CYP88D6 between salt-stressed and control groups [means ± standard deviation (SD); *p < 0.05, **p < 0.01, and ***p < 0.001].

The potential differential expression of 11 crucial genes encoding key proteins in the biosynthesis of glycyrrhizin and flavonoids identified through our analysis was validated in salt-treated and untreated licorice cultivars using qRT-PCR (Figure 6 and Supplementary Table 1). The expression trends of PAL (Glyur000163s00011138), 4CL (Glyur000051s00003417), CHS (Glyur000051s00003428), CHI (Glyur005711s00045445), CYP93C (Glyur000721s00027409), HIDH (Glyur000094s00007841), HI4OMT (Glyur000721s00027413), CYP72A154 (Glyur000890s00019071), CYP88D6 (Glyur000572s00019034), and bAS (Glyur001733s00027628) were highly consistent with the transcriptome data, whereas that of a CYP81E1_7 (Glyur000234s00015060) was partially consistent. The results showed that key rate-limiting genes were significantly upregulated in salt-stressed licorice at the transcriptional level.

### Transcription Factor Analysis

Transcription factors (TFs) that regulate metabolism and response to biotic and abiotic stresses in plants have been identified (Ng et al., 2018). The R2R3-MYB and basic helix–loop–helix (bHLH) family are key factors in the regulatory networks of flavonoid biosynthesis (Xu et al., 2015; Li H. et al., 2019). In the present study, 27 MYB and 14 bHLH were identified to display differential expressions in the two comparison groups (Supplementary Tables 8, 9). Of these genes, *AtMYB111* and *AtMYB11*belong to subgroup 7 of the R2R3-MYBs in *Arabidopsis*, which enhances the level of flavonol biosynthesis in all tissues (Li B. Z. et al., 2019). Two DEGs (*Glyur000211s00017646* and *Glyur000237s00014382*) were successfully mapped to *AtMYB111* and *AtMYB11* and they were increased by 5.75-fold and 1.49-fold (Log2FC value), respectively, following NaCl treatment. Moreover, AtMYB20 enhanced the salt tolerance (Cui et al., 2013), whereas AtMYB73 was a negative regulator of the response to salt stress (Kim et al., 2013). Interestingly, two downregulated genes (*Glyur000023s00004890* and *Glyur000975s00029570*) were identified as *AtMYB73*. However, the TF AtMYB20 (Glyur000730s00036461) showed a high expression level in salt-stressed licorice, indicating that NaCl exerted molecular effects on licorice and activated the response to stress. Furthermore, most bHLHs accepted as essential TFs involved in regulating flavonoid biosynthesis were also upregulated, especially the following genes, *Glyur000099s00014930*, *Glyur000121s00009734*, *Glyur000360s00014611*, and *Glyur006160s00046644* (Supplementary Table 9), which share a similar functional domain with *R2R3-MYB*.

## Discussion

Previous studies have provided evidence indicating that suitable environmental stress such as salinity and drought would increase the accumulation of glycyrrhizin and several flavonoids (Xu et al., 2016; Wang C. C. et al., 2020). However, the molecular mechanisms underlying the biosynthesis of these constituents have not been clarified, which seriously hinders the extensive and effective application of licorice and the cultivation of high-quality plants. In this work, we cultivated licorice seedlings in a simulated salt environment treated with 50 mM NaCl and then performed an integrated transcriptomic and metabolite profiling analysis. The metabolite analysis identified 121 chemicals consisting of 83 flavonoids, 14 triterpenoids, 22 coumarin, and 2 others, among which flavonoids and triterpenoids showed the highest chemical proportion and primarily contributed to the bioactive benefits of licorice. Therefore, we chose these two types of compounds for correlation experiments in the further transcriptomic study.

Flavonoids have properties such as the typical reactive oxygen species scavenging, which highlights their significance under stress conditions. Furthermore, various stress conditions have also been shown to alter the expression of flavonoid biosynthetic genes (Kubra et al., 2021). DEGs are involved in the synthesis of flavonoids from steps involving the formation of chemical scaffolds to subsequent modifications (Nabavi et al., 2020; Wang C. C. et al., 2020). Enzymes involved early in the flavonoid biosynthesis pathway include PAL, CHS, and CHI. Phenylpropanoid biosynthesis begins with the *PAL* gene, an important factor for development and resistance to abiotic stresses in plants that catalyzes L-phenylalanine to *trans-*cinnamic acid. Previous studies suggested that mutant PALs can influence the phenolic content in plants (Nigro et al., 2017; Zhang et al., 2019). The involvement of CHS has been well documented in fruit quality, regulation of flower color, response to environmental stress, and most importantly, in the production and accumulation of flavonoids in plants (El Euch et al., 1998). Overexpression or inactivation of the *CHI* gene has been shown to lead to a remarkable change in flavonoid content (Muir et al., 2001; Kim et al., 2004). These biosynthetic genes and the late pathway enzymes including F3H, FLS, C4H, and 4CL were significantly upregulated after salt treatment. Furthermore, the corresponding glycosides of the flavonoids including chalcone, flavone, flavanone, and isoflavone glycosides exhibited similar expression patterns to those of the DEGs (Figure 4B and Supplementary Figure 4).

Similar to flavonoids, triterpenoid saponins also facilitate the adaptation of plants to UV-B and drought or salt-stress conditions (Zhang et al., 2018). The triterpenoid saponins in licorice are mainly glycyrrhizic acid-related derivatives of the oleanane type, starting from the mevalonic acid pathway of the terpene backbone. The results showed that DEGs of structural triterpenoids including *HMGR*, *GGPS*, *FDLT1*, *SQLE*, and *bAS* was remarkably upregulated. Furthermore, in the salt-stressed group, the expression levels of *CYP* DEGs, which are postmodification enzymes of triterpenoids, were consistent with those of the structural DEGs. The contents of most terpenoids were higher in the salt-stressed group than they were in the control group. Moreover, the terpenoid compounds possessed one to three glycosyl groups except for glycyrrhetinic and glabric acid, which suggests that postmodification glycosyltransferase may be involved in triterpenoid saponin synthesis and contribute to the diversity of triterpenoids in licorice.

On the basis of the chemical features of flavonoid glycosides and triterpenoid saponins, we focused on the expression changes of UGT, which have also been demonstrated to regulate the synthesis of various secondary metabolites and resistance to environmental stress (Liang et al., 2015). The UGT family is a large superfamily of enzymes. The screening methods, experimental materials, and even the content of the databases were different in each research study, and the identified licorice UGTs were different (Huang et al., 2019). For example, with the biosynthesis pathway of glycyrrhizin, multiple different *UGT* genes such as *GuUGAT* (Xu et al., 2016), *UGT73P12* (Nomura et al., 2019), *GuCSyGT* (Chung et al., 2020), and *GuGT14 (UGT73F15)* (Chen et al., 2019) were obtained through a single step involving the conversion of glycyrrhetinic acid to glycyrrhizin.

Phenotypic record search, biochemical analysis, and tracking the determination of bioactive constituents conducted in the transcriptomic research in the present study identified 50 *UGT* genes as DEGs (Supplementary Table 10). Then, the cluster analysis showed that out of all the glycosyltransferases, 41 putative genes were remarkably increased under NaCl stress, as expected (Supplementary Figure 5). Among them, Glyur000289s00018722, Glyur000121s00009707, and Glyur002678s00045101 were significantly upregulated at both the protein and gene expression levels (Supplementary Figure 6; Wang C. C. et al., 2020). Furthermore, Glyur000289s00018722 and Glyur000121s00009707 were coexpressed with key DEGs including *bAS*, *HMGCR*, *FLS*, *CYP88D6*, and *CYP72A154*, which are responsible for flavonoid and saponin biosynthesis.

The results of this study imply that glycosyltransferase might be crucial in the downstream modification of flavonoids and triterpenoids, leading to an increase in liquiritin, isoliquiritin, neoliquiritin, isoliquiritin apioside, and other flavonoid glycosides (Seki et al., 2015). The compounds identified in this analysis have been proven to possess diverse bioactivities and mediate the pharmacological activity of licorice (Kren and Martínková, 2001). In addition, our findings explain the inconsistency of the overexpression of upstream genes and the evident decrease in flavonoid precursors.

## Conclusion

Most of the chemically diverse flavonoids and triterpenoids in licorice have been widely characterized for a long time (Song et al., 2017). The genes involved in the synthesis of these diverse molecules have also been comprehensively identified following the completion of the draft genome (Yu et al., 2015). In this work, the correlation analysis of transcriptome and metabolite profiling revealed that the expression levels of structural and modified genes and significant TFs were closely associated with the accumulation of flavonoids, triterpenoids, and especially their corresponding glycosides. Isolation and characterization of these crucial DEGs in licorice will facilitate the identification of molecular markers for breeding research. Furthermore, *Glyur000289s00018722* and *Glyur000121s00009707* were identified as the most promising *UGT* genes for further developing breeding strategies in licorice. The patterns of the regulation of bioactive constituent expression were also generally in accordance with the results of our previous studies (Wang C. et al., 2020). We expected that our work on licorice chemical constituents will not only contribute to facilitating the breeding of high-quality cultivated products but will also be beneficial in enhancing the understanding of the quality of other herbal medicines. To further study the effects of salt treatments on the accumulation of glycosides in licorice, associated glycosyltransferase might be a promising focus that is worthy of additional in-depth study.

## Data Availability Statement

The datasets presented in this study can be found in online repositories. The names of the repository/repositories and accession number(s) can be found below: NCBI SRA BioProject, accession no: PRJDB3943 (https://www.ncbi.nlm.nih.gov/bioproject/PRJDB3943).

## Author Contributions

CW, SL, and XL conceived and designed the experiments. LC, ZC, JLC, JL, and CC performed the experiments. ZL, LZ, JC, MT, LW, and YM analyzed the data and drafted the manuscript. All authors have read and agreed to the version of the manuscript to be published.

## Conflict of Interest

The authors declare that the research was conducted in the absence of any commercial or financial relationships that could be construed as a potential conflict of interest.

## Publisher’s Note

All claims expressed in this article are solely those of the authors and do not necessarily represent those of their affiliated organizations, or those of the publisher, the editors and the reviewers. Any product that may be evaluated in this article, or claim that may be made by its manufacturer, is not guaranteed or endorsed by the publisher.

## Footnotes

1
https://www.ncbi.nlm.nih.gov/bioproject/PRJDB3943

2
http://cole-trapnell-lab.github.io/cufflinks/

3
http://www.geneontology.org/

4
http://www.genome.jp/kegg/

5
http://deweylab.biostat.wisc.edu/rsem/

6
http://bioinfo.ut.ee/primer3-0.4.0/

## Supplementary Material

Supplementary Figure 1 Morphological and phenotypic profiles of four licorice seedling groups 50 days after treatment with or without NaCl.

Supplementary Figure 2 Representative total ion chromatograms (TIC) in positive mode for **(A)** salt-stressed and **(B)** control licorice.

Supplementary Figure 3 Correlation heatmap of salt-stressed and control groups.

Supplementary Figure 4 Heatmap of metabolite profile for 121 compounds identified in two groups of licorice.

Supplementary Figure 5 Expression profiles of differentially expressed genes (DEGs) of UDP-glycosyltransferases (*UGT*) in licorice.

Supplementary Figure 6 Heat map of DEGs and differentially expressed proteins (DEPs, genes, and proteins of two key UGTs are marked with a red frame.

Supplementary Table 1 Primers for qRT-PCR.

Supplementary Table 2 Identification of 121 compounds in licorice.

Supplementary Table 3 Peak area of 121 compounds in licorice.

Supplementary Table 4 Summary statistics of RNA-seq results of licorice.

Supplementary Table 5 DEGs_detail_Control_VS_Salt.

Supplementary Table 6 DEGs for flavonoid analysis.

Supplementary Table 7 DEGs for triterpenoid analysis.

Supplementary Table 8 DEGs for transcription factor MYB family.

Supplementary Table 9 DEGs for transcription factor bHLH family.

Supplementary Table 10 DEGs for UGT analysis.
