# Supplementary material for: Metabolite Profiling and Transcriptome Analysis Explains Difference in Accumulation of Bioactive Constituents in Licorice (Glycyrrhiza uralensis) Under Salt Stress
Source: Front Plant Sci. 2021 Oct 7;12:727882. doi: 10.3389/fpls.2021.727882 (PMC8529186; doi:10.3389/fpls.2021.727882)
Supplement: Supplementary Figure 1 — Morphological and phenotypic profiles of four licorice seedling groups 50 days after treatment with or without NaCl. [file Data_Sheet_1.zip › Supplementary Figures S1-S6.DOCX]

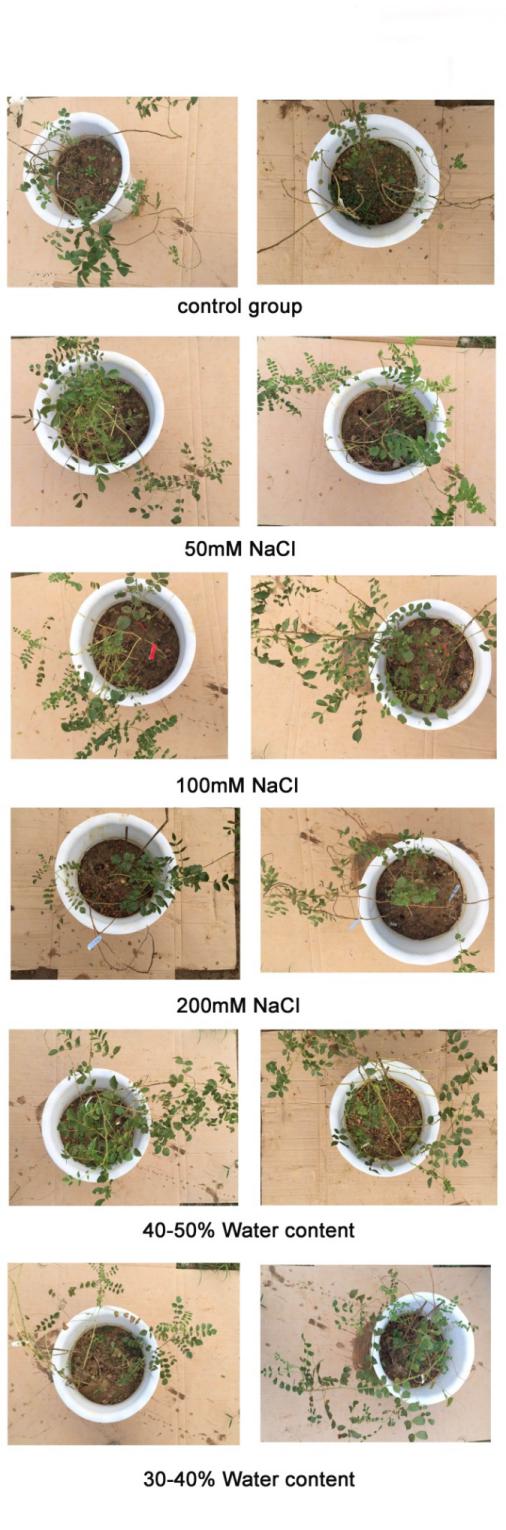


**Figure S1.** Morphological and phenotypic profiles of four licorice seedling groups 50 days after treatment with or without NaCl.


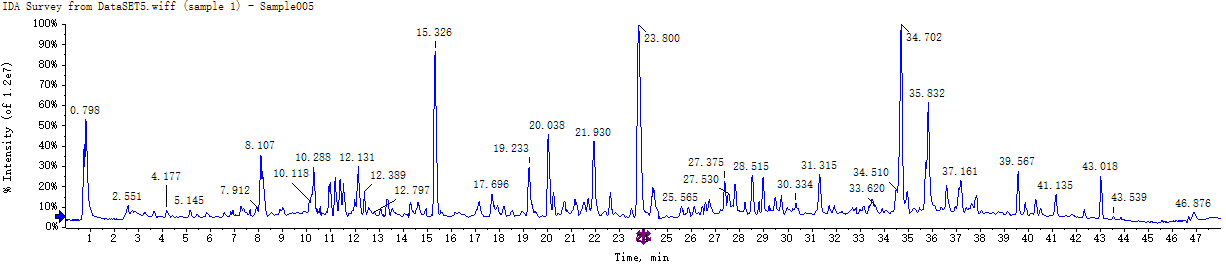


A


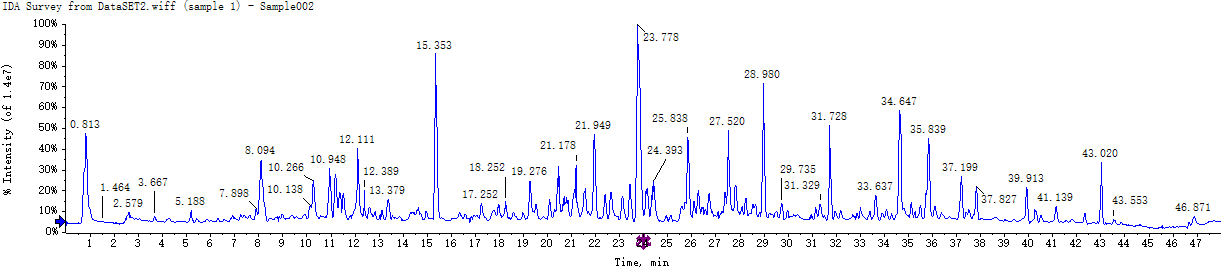


B

**Figure S2.** Representative total ion chromatograms (TIC) in positive mode for (A) salt-stressed and (B) control licorice.


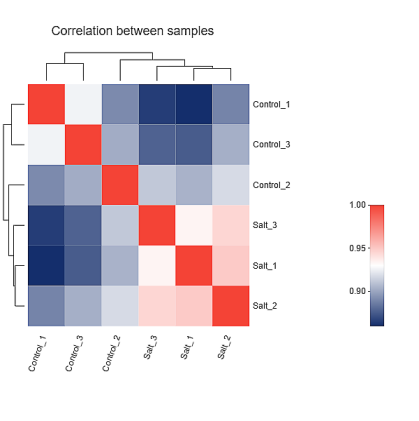


**Figure S3.** Correlation heatmap of salt-stressed and control groups.


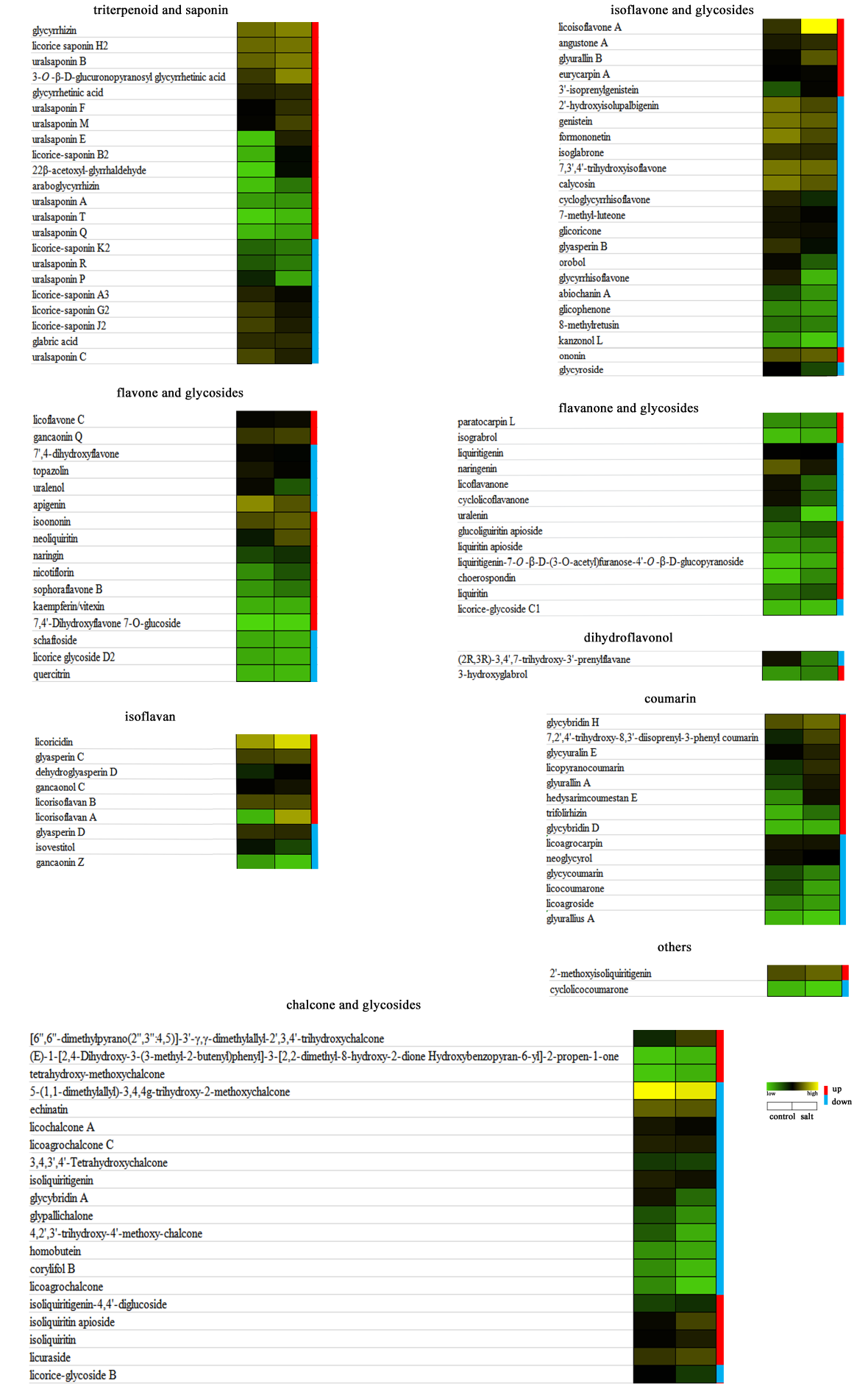


**Figure S4.** Heatmap of metabolite profile for 121 compounds identified in two groups of licorice.


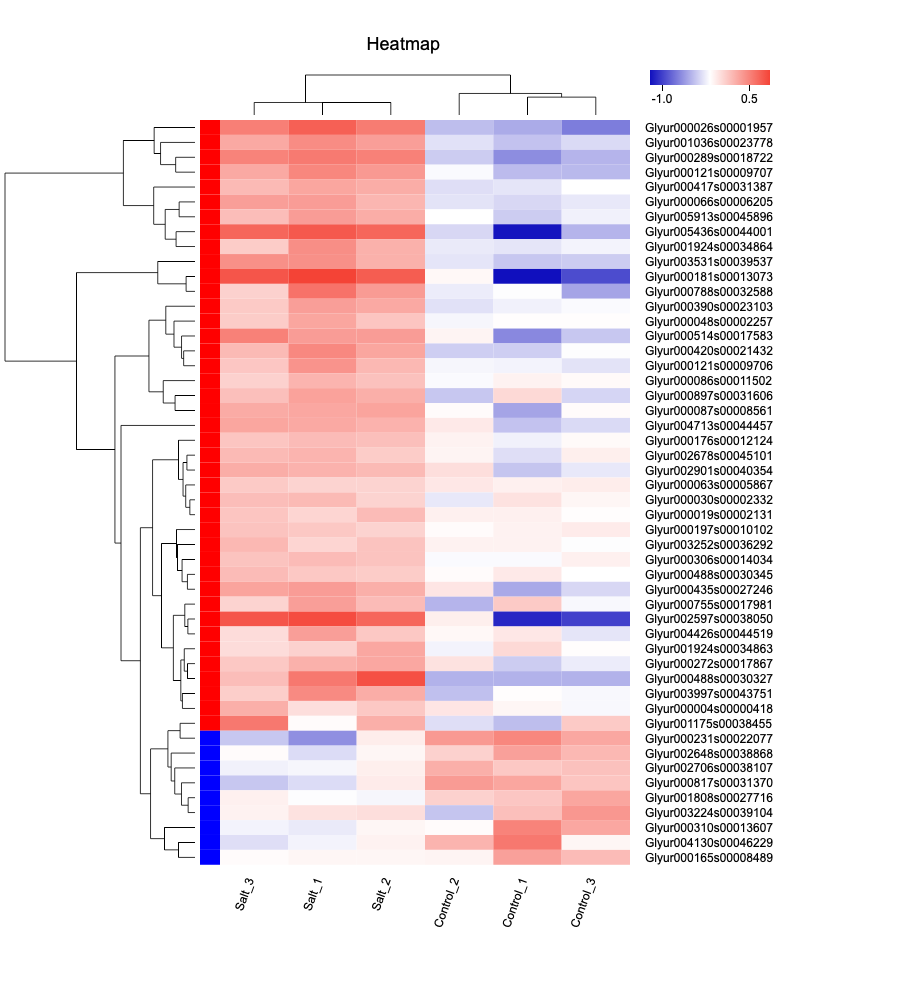


**Figure S5.** Expression profiles of differentially expressed genes (DEGs) of UDP-glycosyltransferases (*UGT*) in licorice


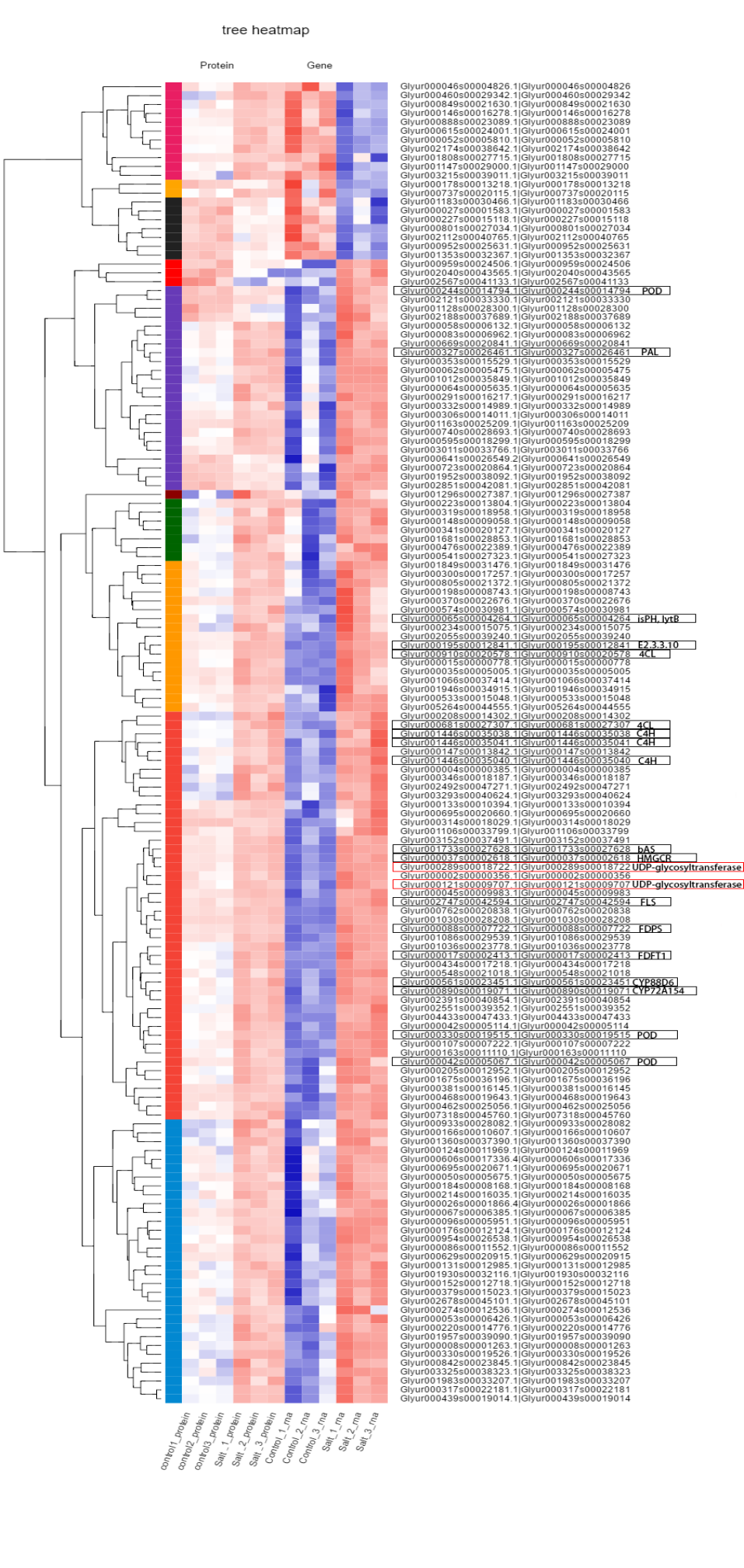


**Figure S6.** Heat map of DEGs and differentially expressed proteins (DEPs, genes and proteins of two key UGTs are marked with red frame
