# Supplementary material for: Metabolite Profiling and Transcriptome Analysis Explains Difference in Accumulation of Bioactive Constituents in Licorice (Glycyrrhiza uralensis) Under Salt Stress
Source: Front Plant Sci. 2021 Oct 7;12:727882. doi: 10.3389/fpls.2021.727882 (PMC8529186; doi:10.3389/fpls.2021.727882)
Supplement: Supplementary Figure 1 — Morphological and phenotypic profiles of four licorice seedling groups 50 days after treatment with or without NaCl. [file Data_Sheet_1.zip › Supplementary Table S1-S3 .DOCX]

**Table S1** Primers for qRT-PCR

| Target gene | Primer | 5’----3’ |
| --- | --- | --- |
| PAL | PAL-F | AGGCTACTCAGGCATCAGA |
|  | PAL-R | CAAACCAGCAATGTAAGAAA |
| 4CL | 4CL-F | GAGTTAGTGTTGCGATGGTG |
|  | 4CL-R | ATCCCGTATCCCTGTCCC |
| CHS | CHS-F | TGGATGCTAGGCAAGACA |
|  | CHS-R | TGGATGCTAGGCAAGACA |
| CHI | CHI-F | CGGGAGCAGCACCAGTAA |
|  | CHI-R | CCGCCGAGGAAATAAGAC |
| CYP93C | CYP93C-F | GGGTCAACATTTCCAACT |
|  | CYP93C-R | CTTCAATATCTTTCCCTGTG |
| HIDH | HIDH-F | GGAGGAGCATGAGCATAG |
|  | HIDH-R | GAGCACCCAAGTGTAGCC |
| HI4OMT | HI4OMT-F | GTTGTGGGTAACTTGTCTG |
|  | HI4OMT-R | GATAATCACCTTCCCTTCT |
| bAS | bAS-F | AGAGAGACCCTGCTCCTCTTCACCGA |
|  | bAS-R | TGGAAGGCAATGGAACCCGTCTACGA |
| CYP81E1_7 | CYP81E1_7-F | GGAGAAGGCTTGGCTATT |
|  | CYP81E1_7-R | TGACTGGACGTGATTTACAC |
| CYP72A154 | CYP72A154-F | GAACACGCTGTGGCTGAG |
|  | CYP72A154-R | GATGGGCAAGGGAGAAGA |
| CYP88D6 | CYP88D6-F | ACCACCATCTTCGTAGCC |
|  | CYP88D6-R | GTGCCTTGAGTGCTTTAT |
| β-actin | β-actin-F | GGCTGTGTTTCCCAGTATTGT |
|  | β-actin-R | CATAACCCTCATAGATTGGCAC |

**Table S2** Identification of 121 compounds in licorice

| No | t**_R_/(min)** | Putative compound | Molecular formula | ppm | Measured mass (***m/z*)** | MS**^2^fragments** |
| --- | --- | --- | --- | --- | --- | --- |
| 1 | 5.94 | nicotiflorin | C_27_H_30_O_15_ | 3.1 | 595.1669(+) | 463.1047[M+H-(Rha-CH_2_)]+, 415.0799[M+H-(Rha-CH_2_)-CH_2_O-OH]+, 375.0852[M+H-(Rha-CH_2_)-C_6_H_5_O-OH]+ |
| 2 | 7.24 | sophoraflavone B | C_21_H_20_O_9_ | 2 | 417.1180(+) | 255.0656[M+H-Glc]+ |
| 3 | 7.26 | 7,4'-Dihydroxyflavone-7-*O*-glucoside | C_21_H_20_O_9_ | 2 | 417.1191(+) | 255.0651[M+H-Glc]+ |
| 4 | 7.64 | isoliquiritigenin-4,4'-diglucoside | C_27_H_32_O_14_ | -0.7 | 581.1864(+) | 419.1364[M+H-Glc]+, 257.0806[M+H-Glc-C_9_H_6_O_3_]+ |
| 5 | 7.66 | glucoliguiritin apioside | C_32_H_40_O_18_ | 3.3 | 713.2313(+) | 257.0808[M+H-2Glc-Api]+ |
| 6 | 7.71 | naringin * | C_27_H_32_O_14_ | -0.5 | 581.1868(+) | 419.1340[M+H-Glc]+,257.0810[M+H-2Glc-H_2_O]+ |
| 7 | 7.89 | trifolirhizin | C_22_H_22_O_10_ | 2.4 | 447.1295(+) | 285.0751[M+H-Glc]+, 269.0630[M+H-Glc-O]+, 137.0233[M+H-Glc-C_9_H_7_O]+ |
| 8 | 8.14 | neoliquiritin * | C_21_H_22_O_9_ | 4.4 | 419.1344(+) | 257.0804[M+H-Glc]+, 239.0701[M+H-Glc-H_2_O]+, 137.0229[M+H-Glc-H_2_O-C_7_H_3_O]+, 119.0479[M+H-Glc-2H_2_O-C_7_H_3_O]+ |
| 9 | 8.16 | liquiritin apioside * | C_26_H_30_O_13_ | 4.3 | 551.1774(+) | 257.0809[M+H-Glc-Ara]+, 239.0695[M+H-Glc-Ara-H_2_O]+, 137.0223[M+H-Glc-Ara-H_2_O]+ |
| 10 | 8.78 | orobol | C_15_H_10_O_6_ | 1.1 | 287.0550(+) | 287.0459, 153.0184[M+H-C_8_H_6_O_2_]+ |
| 11 | 8.79 | liquiritin * | C_21_H_22_O_9_ | 4.4 | 419.1344(+) | 257.0804[M+H-Glc]+, 239.0698[M+H-Glc-H_2_O]+, 137.0230[M+H-Glc-H_2_O-C_8_H_7_]+ |
| 12 | 9.90 | quercitrin | C_21_H_20_O_11_ | 0.5 | 449.1078(+) | 287.0916[M+H-Glc]+, 269.0809[M+H-Glc-H_2_O]+ |
| 13 | 9.93 | schaftoside | C_26_H_28_O_14_ | 0.9 | 565.1551(+) | 565.1559,271.0602[M+H-Glc-Ara]+ |
| 14 | 10.00 | choerospondin | C_21_H_22_O_10_ | 0.6 | 435.1294(+) | 273.0740[M+H-Glc]+,153.0179[M+H-Glc-C_8_H_8_O]+ |
| 15 | 10.09 | kaempferin/vitexin | C_21_H_20_O_10_ | 2.4 | 433.1143(+) | 271.0592[M+H-Rha-OH]+/271.0650[M+H-Glc]+ |
| 16 | 11.44 | licoagroside | C_22_H_20_O_10_ | 0.3 | 445.11292 | 283.0606[M+H-Glc]+, 253.0491[M+H-Glc-CH_3_O]+ |
| 17 | 11.53 | licuraside | C_26_H_30_O_13_ | -0.6 | 551.1759(+) | 257.0800[M+H-Glc-Api]+,257.0800[M+H-Glc-Api-C_6_H_4_O-H_2_O]+, |
| 18 | 11.55 | isoliquiritin apioside * | C_26_H_30_O_13_ | -0.6 | 551.1761(+) | 257.0804[M+H-Glc-Api]+,137.0232[M+H-Glc-Api-H_2_O-C_8_H_6_]+ |
| 19 | 11.60 | glycyroside | C_27_H_30_O_13_ | -0.1 | 563.1759(+) | 269.0809[M+H-Glc-Api]+ |
| 20 | 11.81 | liquiritigenin-7-*O*-β-D-(3-*O*-acetyl)furanose-4'-*O*-β-D-glucopyranoside | C_28_H_32_O_14_ | -0.6 | 593.18648(+) | 299.0897[M+H-C_14_H_14_O_7_]+,269.0807[M+H-C_14_H_14_O_7_-CH_2_O]+ |
| 21 | 11.85 | 7',4-dihydroxyflavone | C_15_H_10_O_4_ | 0.6 | 255.0651(+) | 255.0649,137.0238[M+H-C_8_H_6_O]+,91.0562[M+H-C_8_H_6_O-H_2_O-CO_2_]+, |
| 22 | 11.97 | isoliquiritin * | C_21_H_22_O_9_ | 4.4 | 419.1351(+) | 257.0804[M+H-Glc]+, 239.0698[M+H-Glc-H_2_O]+, 137.0230[M+H-Glc-H_2_O-C_8_H_6_]+ |
| 23 | 12.15 | isoononin | C_22_H_22_O_9_ | -0.1 | 431.1336(+) | 269.0794[M+H-Glc]+,237.0541[M+H-Glc-OCH_3_]+ |
| 24 | 12.18 | ononin | C_22_H_22_O_9_ | 4.2 | 431.1353(+) | 269.0812[M+H-Glc]+, 237.0552[M+H-Glc-OCH_3_]+ |
| 25 | 12.71 | homobutein | C_16_H_14_O_5_ | 1 | 287.0914(+) | 287.0901,237.0541[M+H-C_9_H_8_O_2_-H_2_O]+ |
| 26 | 13.03 | liquiritigenin * | C_15_H_12_O_4_ | 2 | 257.0816(+) | 165.0698[M+H-C_6_H_5_O]+, 137.0234[M+H-C_6_H_5_O-C_2_H_3_]+ |
| 27 | 13.34 | licorice-glycoside B | C_35_H_36_O_15_ | -0.8 | 697.2127(+) | 279.0862[M+H-C_21_H_22_O_9_]+,147.0438[M+H-C_21_H_22_O_9_-Api]+ |
| 28 | 14.42 | 3,4,3',4'-Tetrahydroxychalcone | C_15_H_12_O_5_ | 0.8 | 273.0757(+) | 137.0238[M+H-C_8_H_7_O_2_]+,121.0291[M+H-C_8_H_7_O_2_-OH]+ |
| 29 | 16.28 | uralsaponin T | C_48_H_74_O_19_ | 6.3 | 955.4974(+) | 603.4213[M+H-2Glucuronic acid]+, 585.4149[M+H-2Glucuronic acid-H_2_O]+, 439.3575[M+H-2Glucuronic acid-H_2_O-Rha]+ |
| 30 | 16.38 | licorice glycoside D2 | C_35_H_36_O_15_ | 5.2 | 697.2167(+) | 257.0787[M+H-C_20_H_25_O_11_]+, 215.0696[M+H-C_20_H_25_O_11_-C_2_HO]+, 137.0230[M+H-C_20_H_25_O_11_-C_2_HO-C_6_H_6_]+ |
| 31 | 16.59 | licorice-glycoside C1 | C_36_H_38_O_16_ | -0.2 | 727.2232(+) | 727.2321,257.0801[M+H-C_21_H_27_O_12_]+ |
| 32 | 17.26 | licorice-saponin A3 | C_48_H_72_O_22_ | -0.4 | 1001.4638(+) | 1001.4559,825.4251[M+H-Glucuronic acid]+，649.3939[M+H-2Glucuronic acid]+，631.3829[M+H-2Glucuronic acid-H_2_O]+，469.3306[M+H-2Glucuronic acid-H_2_O-Glc]+，451.3211[M+H-2Glucuronic acid-2H_2_O-Glc]+ |
| 33 | 17.78 | uralsaponin F | C_44_H_64_O_19_ | 6.1 | 897.4169(+) | 545.3469[M+H-2Glucuronic acid]+, 527.3351[M+H-2Glucuronic acid-H_2_O]+, 497.3223[M+H-2Glucuronic acid-H_2_O-CH_2_O]+ |
| 34 | 18.01 | araboglycyrrhizin | C_41_H_62_O_14_ | -2 | 779.4212(+) | 779.4595，439.3522[M+H-Glucuronic acid-Ara-CH_3_]+ |
| 35 | 20.08 | uralsaponin M | C_44_H_64_O_18_ | -0.7 | 881.4165(+) | 705.3838[M+H-Glucuronic acid]+,529.3487[M+H-2Glucuronic acid]+,511.3407[M+H-2Glucuronic acid-H_2_O]+ |
| 36 | 20.17 | uralsaponin E | C_42_H_60_O_17_ | -0.1 | 837.3903(+) | 837.3899,485.3264[M+H-2Glucuronic acid]+,467.3161[M+H-2Glucuronic acid-H_2_O]+,449.3044[M+H-2Glucuronic acid-2H_2_O]+ |
| 37 | 20.69 | uralsaponin R | C_48_H_74_O_20_ | 6.9 | 971.4842(+) | 439.3568[M+H-Glc-Rha-Glucuronic acid-COOH]+ |
| 38 | 20.69 | isoliquiritigenin | C_15_H_12_O_4_ | 2 | 257.1100(+) | 137.0228[M+H-C_8_H_8_O]+ |
| 39 | 21.16 | formononetin * | C_16_H_12_O_4_ | 3.4 | 269.0811(+) | 253.0503[M+H-CH_3_]+, 237.0555[M+H-CH_3_-O]+, 213.0914[M+H-CH_3_-O-C_2_H]+, 137.0236[M+H-CH_3_-O-C_2_H-C_6_H_4_]+ |
| 40 | 21.24 | uralsaponin Q | C_47_H_72_O_19_ | 7.2 | 941.4817(+) | 647.3736[M+H-Rha-Xyl]+, 471.3449[M+H-Rha-Xyl-Glucuronic acid]+, 453.3351[M+H-Rha-Xyl-Glucuronic acid-H_2_O]+ |
| 41 | 21.28 | eurycarpin A | C_20_H_18_O_5_ | 0.4 | 339.1227(+) | 339.1223, 283.0600[M+H-C_4_H_7_]+ |
| 42 | 21.30 | uralenin | C_20_H_20_O_6_ | 0 | 357.1332(+) | 339.1223[M+H-H_2_O]+, 283.0599[M+H-H_2_O-C_4_H_7_]+,165.0183[M+H-H_2_O-C_4_H_7_-C_8_H_6_O]+ |
| 43 | 21.59 | naringenin | C_15_H_12_O_5_ | 0.8 | 273.07575(+) | 137.0599[M+H-C_7_H_4_O_3_]+,123.0446[M+H-C_8_H_4_O_3_]+ |
| 44 | 21.90 | glabric acid | C_30_H_46_O_5_ | 3.7 | 487.3451(+) | 469.3352[M+H-H_2_O]+, 451.3213[M+H-2H_2_O]+, 317.2475[M+H-COOH-OH-C_10_H_14_]+ |
| 45 | 22.05 | 22β-acetoxyl-glyrrhaldehyde | C_44_H_64_O_17_ | -0.4 | 865.42163(+) | 865.4074,689.3871[M+H-Glucuronic acid]+,513.3569[M+H-2Glucuronic acid]+,495.3425[M+H-2Glucuronic acid-H_2_O]+ |
| 46 | 22.59 | licorice-saponin G2 | C_42_H_62_O_17_ | -0.9 | 839.40598(+) | 839.4016,487.3408[M+H-2Glucuronic acid]+,469.3292[M+H-2Glucuronic acid-H_2_O]+,451.3197[M+H-2Glucuronic acid-2H_2_O]+ |
| 47 | 22.70 | licorice-saponin K2 | C_42_H_62_O_16_ | -0.8 | 823.41106(+) | 647.3763[M+H-Glucuronic acid]+,471.3444[M+H-2Glucuronic acid]+,453.3359[M+H-2Glucuronic acid-2H_2_O]+ |
| 48 | 22.92 | abiochanin A | C_16_H_12_O_5_ | 0.6 | 285.0757(+) | 175.0386[M+H-C_6_H_5_O_2_]+,147.0438[M+H-C_6_H_5_O_2_-CO]+ |
| 49 | 22.94 | glypallichalone | C_17_H_16_O_4_ | 0.2 | 285.1121(+) | 255.0655[M+H-CH_3_O]+,151.0394[M+H-CH_3_O-C7H4O]+,123.0448[M+H-CH_3_O-C_7_H_4_O-C_2_H_3_]+ |
| 50 | 23.71 | uralsaponin B | C_42_H_62_O_16_ | -0.8 | 823.4110(+) | 647.3776[M+H-Glucuronic acid]+,471.3465[M+H-2Glucuronic acid]+,453.3339[M+H-2Glucuronic acid-2H_2_O]+ |
| 51 | 23.76 | licorice saponin H2 | C_42_H_62_O_16_ | -0.8 | 823.4113(+) | 647.3757[M+H-Glucuronic acid]+,471.3452[M+H-2Glucuronic acid]+,453.3332[M+H-2Glucuronic acid-2H_2_O]+ |
| 52 | 23.81 | glycyrrhizin * | C_42_H_62_O_16_ | 3.6 | 823.4146(+) | 471.3448[M+H-2Glucuronic acid]+, 453.3347[M+H-2Glucuronic acid-H_2_O]+, 407.3286[M+H-2Glucuronic acid-COOH]+ |
| 53 | 23.97 | 3-*O*-β-D-glucuronopyranosyl glycyrrhetinic acid | C_36_H_54_O_10_ | 4.7 | 647.3756(+) | 453.3352[M+H-Glucuronic acid-H_2_O]+, 435.3242[M+H-Glucuronic acid-2H_2_O]+, 407.3291[M+H-Glucuronic acid-H_2_O-COOH]+ |
| 54 | 24.33 | echinatin * | C_16_H_14_O_4_ | 0.4 | 271.0964(+) | 137.0590[M+H-C_7_H_5_O_2_-CH_2_]+ |
| 55 | 24.36 | 7,3',4'-trihydroxyisoflavone | C_15_H_10_O_5_ | 2.1 | 271.0601(+) | 161.0595[M+H-C_6_H_5_O_2_]+,137.0591[M+H-C_6_H_5_O_2_-C_2_H]+ |
| 56 | 24.38 | 2'-methoxyisoliquiritigenin | C_16_H_14_O_4_ | 0.4 | 271.0963(+) | 137.0591[M+H-C_8_H_7_O-CH_3_]+,109.0656[M+H-C_8_H_7_O-CH_3_-CO]+ |
| 57 | 24.42 | apigenin | C_15_H_10_O_5_ | 2.1 | 271.0958(+) | 137.0590[M+H-C_8_H_6_O_2_]+,109.0654[M+H-C_8_H_6_O_2_-CO]+ |
| 58 | 24.44 | calycosin | C_15_H_10_O_5_ | 2.1 | 271.0962(+) | 161.0595[M+H-C_6_H_5_O_2_]+,137.0590[M+H-C_6_H_5_O_2_-C_2_H]+ |
| 59 | 24.48 | genistein | C_15_H_10_O_5_ | 2.1 | 271.0961(+) | 137.0593[M+H-C_8_H_6_O_2_]+,109.0659[M+H-C_8_H_6_O_2_-CO]+ |
| 60 | 24.59 | glycyuralin E | C_21_H_22_O_6_ | 4.1 | 371.1501(+) | 297.0750[M+H-C_4_H_8_-H_2_O]+, 165.0535[M+H-C_12_H_13_O_2_-H_2_O]+, 135.0427[M+H-C_13_H_13_O_2_-2H_2_O]+ |
| 61 | 24.94 | isovestitol | C_16_H_16_O_4_ | 1.3 | 273.1121(+) | 137.0238[M+H-C_8_H_9_O_2_]+,121.0293[M+H-C_8_H_9_O_2_-O]+ |
| 62 | 25.59 | licorice-saponin B2 | C_42_H_64_O_15_ | -0.5 | 809.4318(+) | 809.4299,633.3996[M+H-Glucuronic acid]+,457.3673[M+H-2Glucuronic acid]+,439.3575[M+H-2Glucuronic acid-H_2_O]+ |
| 63 | 25.70 | glyurallius A | C_20_H_18_O_4_ | 0.3 | 323.1277(+) | 323.1270,267.0652[M+H-C_4_H_7_]+,239.0720[M+H-C_4_H_7_-H_2_O]+ |
| 64 | 26.74 | uralsaponin C | C_42_H_64_O_16_ | -1.8 | 825.4267(+) | 825.4229,631.3848[M+H-Glucuronic acid-H_2_O]+,455.3511[M+H-2Glucuronic acid-H_2_O]+,437.3400[M+H-2Glucuronic acid-2H_2_O]+ |
| 65 | 26.76 | licorice-saponin J2 | C_42_H_64_O_16_ | -1.8 | 825.4253(+) | 825.4228,631.3824[M+H-Glucuronic acid-H_2_O]+,455.3514[M+H-2Glucuronic acid-H_2_O]+,437.3410[M+H-2Glucuronic acid-2H_2_O]+ |
| 66 | 27.28 | uralsaponin P | C_42_H_64_O_16_ | -1.8 | 825.4251(+) | 825.4255,649.3964[M+H-Glucuronic acid-CH_2_]+ |
| 67 | 27.34 | glicophenone | C_20_H_22_O_6_ | 0 | 359.1507(+) | 257.0851[M+H-C_5_H_10_-OCH_3_]+ |
| 68 | 27.39 | tetrahydroxy-methoxychalcone | C_16_H_14_O_6_ | 2.8 | 303.0869(+) | 165.0578[M+H-C_6_H_5_O_2_-CO]+, 135.0417[M+H-C6H5O2-CO-OCH3]+, 107.0505[M+H-C_6_H_5_O_2_-CO-OCH_3_-C_2_H_4_]+ |
| 69 | 27.42 | uralsaponin A | C_42_H_62_O_16_ | -0.8 | 823.4110(+) | 279.0862[M+H-C_21_H_22_O_9_]+,147.0438[M+H-C_21_H_22_O_9_-Api]+ |
| 70 | 27.67 | glyasperin C | C_21_H_24_O_5_ | 2.2 | 357.1713(+) | 283.0676[M+H-C_4_H_8_-H_2_O]+, 165.0555[M+H-C_4_H_8_-H_2_O-C_8_H_6_O]+, 137.0532[M+H-C_4_H_8_-H_2_O-C_8_H_6_O-CO]+, 123.0421[M+H-C_4_H_8_-H_2_O-C_8_H_6_O-CO-CH_2_]+ |
| 71 | 27.72 | glycybridin A | C_20_H_20_O_5_ | 0.2 | 341.1383(+) | 341.1380, 165.0178[M+H-C_11_H_11_O-OH]+ |
| 72 | 27.74 | licoflavanone | C_20_H_20_O_5_ | 0.2 | 341.1385(+) | 341.1382, 285.0751[M+H-C_4_H_7_]+,165.0178[M+H-C_4_H_7_-C_8_H_7_O]+ |
| 73 | 27.77 | (2R,3R)-3,4',7-trihydroxy-3'-prenylflavane | C_20_H_20_O_5_ | 0.7 | 341.1393(+) | 137.0239[M+H-C_13_H_16_O_2_]+ |
| 74 | 27.82 | cyclolicoflavanone | C_20_H_20_O_5_ | 0.2 | 341.1387(+) | 341.1380, 285.0751[M+H-C_4_H_7_]+,165.0178[M+H-C_4_H_7_-C_8_H_6_O]+ |
| 75 | 27.84 | licocoumarone | C_20_H_20_O_5_ | 0.2 | 341.1384(+) | 341.1384, 285.0754[M+H-C_4_H_7_]+,165.0178[M+H-C_4_H_7_-C_7_H_5_O_2_]+ |
| 76 | 27.89 | glycybridin H | C_21_H_24_O_5_ | 2.2 | 357.1341(+) | 301.0685[M+H-C_4_H_8_]+, 283.0772[M+H-C_4_H_8_-H_2_O]+, 179.0343[M+H-C_4_H_8_-H_2_O-C_7_H_4_O]+, 165.5554[M+H-C_4_H_8_-H_2_O-C_8_H_2_O]+, 151.0387[M+H-C_4_H_8_-H_2_O-C_9_H_4_O]+, 139.0398[M+H-C_4_H_8_-H_2_O-C_10_H_4_O]+, 125.0596[M+H-C_4_H_8_-H_2_O-C_10_H_4_O-CH_3_]+, 107.0477[M+H-C_4_H_8_-H_2_O-C_10_H_4_O-OCH_3_]+ |
| 77 | 27.93 | cycloglycyrrhisoflavone | C_20_H_18_O_6_ | 0.3 | 355.1176(+) | 355.1175,299.0556[M+H-C_4_H_8_]+,281.0446[M+H-C_4_H_8_-H_2_O]+,137.0238[M+H-C_4_H_8_-H_2_O-C_8_H_6_O_2_]+ |
| 78 | 28.26 | licoisoflavone A | C_20_H_18_O_6_ | 0.3 | 355.1172(+) | 355.1166,299.0540[M+H-C_4_H_8_]+,287.0539[M+H-C_4_H_8_-C]+,271.0597[M+H-C_4_H_8_-C-OH]+ |
| 79 | 28.58 | 4,2',3'-trihydroxy-4'-methoxy-chalcone | C_20_H_18_O_4_ | 0.3 | 287.1281(+) | 177.0961[M+H-C_6_H_5_O-OH]+,151.0758[M+H-C_6_H_5_O-OH-C_2_H_2_]+ |
| 80 | 28.94 | uralenol | C_20_H_18_O_7_ | -0.1 | 371.1125(+) | 315.0494[M+H-C_4_H_8_]+,137.0238[M+H-C_4_H_8_-C_9_H_6_O_4_]+ |
| 81 | 28.99 | 5-(1,1-dimethylallyl)-3,4,4g-trihydroxy-2-methoxychalcone | C_21_H_22_O_5_ | -0.4 | 355.0044(+) | 355.1161,327.1220[M+H-C_4_H_8_]+,137.0238[M+H-C_2_H_3_]+,179.0329[M+H-C_2_H_3_-C_10_H_6_O_2_]+ |
| 82 | 29.03 | 8-methylretusin | C_17_H_14_O_5_ | 0.3 | 299.0926(+) | 267.0652[M+H-OCH_3_]+, 248.9875[M+H-OCH_3_-H_2_O]+, 159.0507[M+H-OCH_3_-H_2_O-C_6_HO]+ |
| 83 | 29.59 | licoagrocarpin | C_21_H_22_O_4_ | 2.5 | 339.1243(+) | 283.0597[M+H-C_4_H_8_]+, 255.0648[M+H-C_4_H_8_-C-CH_3_]+ |
| 84 | 29.61 | licochalcone A | C_21_H_22_O_4_ | -1.4 | 339.1590(+) | 339.1221,283.0594[M+H-CH_3_O-C_2_H_2_]+,241.0493[M+H-CH_3_O-C_2_H_2_-C_3_H_6_]+ |
| 85 | 29.80 | 3'-isoprenylgenistein | C_20_H_18_O_5_ | 0.4 | 339.1227(+) | 339.1226,283.0599[M+H-C_4_H_7_]+,271.0597[M+H-C_4_H_7_-C]+,255.0641[M+H-C_4_H_7_-C]+ |
| 86 | 30.04 | gancaonol C | C_21_H_22_O_5_ | -0.4 | 355.1543(+) | 355.1543,153.0551[M+H-C_13_H_14_O_2_]+,123.0453[M+H-C_13_H_14_O_2_-CH_2_O]+ |
| 87 | 30.20 | cyclolicocoumarone | C_20_H_20_O_5_ | 0.2 | 341.1383(+) | 341.1385,285.0757[M+H-C_4_H_8_]+,149.0228[M+H-C_4_H_8_-C_8_H_8_O_2_]+ |
| 88 | 30.49 | licoflavone C | C_20_H_18_O_5_ | 0.4 | 339.1228(+) | 283.0590[M+H-C_4_H_8_]+,165.0179[M+H-C_4_H_8_-C_7_H_3_O_2_]+ |
| 89 | 31.01 | corylifol B | C_20_H_20_O_5_ | 0.2 | 341.1383(+) | 285.0762[M+H-C_4_H_8_]+,165.0179[M+H-C_4_H_8_-C_7_H_5_O_2_]+ |
| 90 | 31.18 | glycyrrhisoflavone | C_20_H_18_O_6_ | 0.3 | 355.1176(+) | 299.0541[M+H-C_4_H_8_]+,123.0092[M+H-C_4_H_8_-C_9_H_5_O_4_]+ |
| 91 | 31.30 | licoagrochalcone C | C_21_H_22_O_5_ | -0.4 | 355.1540(+) | 299.0548[M+H-C_4_H_8_]+,165.0171[M+H-C_4_H_8_-C_8_H_6_O_2_]+ |
| 92 | 32.52 | licoagrochalcone | C_20_H_20_O_4_ | 0 | 325.1434(+) | 325.1424,269.0811[M+H-C_4_H_8_]+,149.0231[M+H-C_4_H_8_-C_7_H_4_O-OH]+ |
| 93 | 32.72 | gancaonin Z | C_21_H_24_O_4_ | 0 | 341.1747(+) | 285.1114[M+H-C_4_H_8_]+,149.0595[M+H-C_4_H_8_-C_8_H_8_O_2_]+,137.0603[M+H-C_4_H_8_-C_8_H_8_O_2_-C]+ |
| 94 | 32.72 | glycycoumarin | C_21_H_20_O_6_ | -0.5 | 369.1332(+) | 369.1326,313.0706[M+H-C_4_H_8_]+,295.0602[M+H-C_4_H_8_-H_2_O]+ |
| 95 | 32.88 | isoglabrone | C_20_H_16_O_5_ | 0.5 | 337.1070(+) | 337.1053,295.0600[M+H-C_3_H_6_]+,279.0645[M+H-C_3_H_6_-O]+,165.0706[M+H-C_3_H_6_-O-C_8_H_3_O]+ |
| 96 | 33.05 | glyasperin D | C_22_H_26_O_5_ | 3.4 | 371.1874(+) | 250.9766[M+H-Phenol-H_2_O]+, 219.0994[M+H-Phenol-H_2_O-CH_3_-O]+, 205.1093[M+H-Phenol-H_2_O-CH_3_-O-CH_2_]+ |
| 97 | 33.38 | glicoricone | C_21_H_20_O_6_ | -0.5 | 369.1328(+) | 369.1338,313.0702[M+H-C_4_H_8_]+,295.0602[M+H-C_4_H_8_-H_2_O]+,283.0599[M+H-C_4_H_8_-H_2_O-C]+ |
| 98 | 33.41 | topazolin | C_21_H_20_O_6_ | -0.5 | 369.1332(+) | 369.1334,313.0701[M+H-C_4_H_8_]+,295.0604[M+H-C_4_H_8_-H_2_O]+,283.0586[M+H-C_4_H_8_-H_2_O-C]+,179.0338[M+H-C_4_H_8_-H_2_O-C]+ |
| 99 | 33.43 | 7-methyl-luteone | C_21_H_20_O_6_ | -0.5 | 369.1338(+) | 369.1337,313.0703[M+H-C_4_H_8_]+,295.0603[M+H-C_4_H_8_-H_2_O]+,179.0339[M+H-C_4_H_8_-H_2_O-C_8_H_4_O]+ |
| 100 | 33.72 | glyasperin B | C_21_H_22_O_6_ | 4.1 | 371.1875(+) | 153.0532[M+H-C_13_H_14_O_3_]+, 137.0596[M+H-C_13_H_14_O_3_-O]+, 123.0811[M+H-C_14_H_16_O_3_-O]+ |
| 101 | 34.02 | paratocarpin L | C_25_H_28_O_5_ | -1 | 409.2009(+) | 409.1996,353.1384[M+H-C_4_H_8_]+,297.0751[M+H-2C_4_H_8_]+,269.0818[M+H-2C_4_H_8_-2C]+ |
| 102 | 34.65 | licoricidin | C_26_H_32_O_5_ | 5.9 | 425.0516(+) | 221.1167[M+H-C_13_H_6_O_2_]+, 189.0904[M+H-C_13_H_6_O_2_-CH_2_O]+, 177.0527[M+H-C_13_H_6_O_2_-CH_2_O-C]+, 135.0438[M+H-C_13_H_6_O_2_-CH_2_O-C-C_2_HO]+ |
| 103 | 35.31 | angustone A | C_25_H_26_O_6_ | 7.6 | 423.1841(+) | 367.1175[M+H-C_4_H_8_]+, 311.0556[M+H-2C_4_H_8_]+, 299.0557[M+H-2C_4_H_8_-C]+ |
| 104 | 35.70 | dehydroglyasperin D | C_22_H_24_O_5_ | 0.6 | 369.0243(+) | 299.0912[M+H-C_5_H_9_]+, 285.0758[M+H-C_5_H_9_-CH_3_]+, 163.0390[M+H-C_5_H_9_-CH_3_-C_7_H_6_O_2_]+ |
| 105 | 35.70 | licorisoflavan B | C_26_H_32_O_5_ | -1.7 | 425.2322(+) | 425.1953,369.1326[M+H-C_4_H_8_]+,351.1231[M+H-C_4_H_8_-H_2_O]+ |
| 106 | 36.41 | glycyrrhetinic acid * | C_30_H_46_O_4_ | 3.2 | 471.3471(+) | 453.3368[M+H-H_2_O]+, 435.3267[M+H-2H_2_O]+, 407.3325[M+H-2H_2_O-CO]+ |
| 107 | 36.69 | glyurallin A | C_21_H_20_O_5_ | 0 | 353.1383(+) | 353.1360,297.0759[M+H-C_4_H_8_]+,267.0652[M+H-C_4_H_8_-H_2_O-C]+ |
| 108 | 37.07 | licopyranocoumarin | C_21_H_20_O_7_ | 0.1 | 385.1281(+) | 311.0914[M+H-C_3_H_6_O_2_]+, 283.0956[M+H-C_3_H_6_O_2_-C_2_H_4_]+ |
| 109 | 37.10 | hedysarimcoumestan E | C_17_H_12_O_7_ | 0.4 | 329.0655(+) | 311.0923[M+H-H_2_O]+,137.0601[M+H-H_2_O]+ |
| 110 | 37.22 | neoglycyrol | C_21_H_18_O_6_ | -0.6 | 367.1176(+) | 349.1068[M+H-H_2_O]+,189.0909[M+H-H_2_O-C_9_H_3_O_3_]+ |
| 111 | 37.27 | 2'-hydroxyisolupalbigenin | C_25_H_26_O_6_ | -1.2 | 423.1802(+) | 423.1802,367.1175[M+H-C_4_H_8_]+,349.1066[M+H-C_4_H_8_-H_2_O]+,189.0905[M+H-C_4_H_8_-H_2_O-C_11_H_12_O]+ |
| 112 | 37.43 | 7,2',4'-trihydroxy-8,3'-diisoprenyl-3-phenyl coumarin | C_25_H_26_O_5_ | -1 | 407.1853(+) | 407.1850,351.1226[M+H-C_4_H_8_]+,295.0601[M+H-2C_4_H_8_]+,283.0597[M+H-2C_4_H_8_-C]+ |
| 113 | 37.55 | glyurallin B | C_20_H_18_O_6_ | 0.3 | 423.1794(+) | 423.1816,367.1176[M+H-C_4_H_8_]+,311.0549[M+H-2C_4_H_8_]+,283.0604[M+H-2C_4_H_9_-C]+ |
| 114 | 37.75 | [6'',6''-dimethylpyrano(2'',3'':4,5)]-3'-γ,γ-dimethylallyl-2',3,4'-trihydroxychalcone | C_25_H_26_O_5_ | -1 | 407.2185(+) | 351.1223[M+H-C_4_H_8_]+,295.0596[M+H-C_4_H_8_-C_3_H_3_O]+,177.0177[M+H-C_4_H_8_-C_3_H_3_O-C_7_H_3_O_2_]+ |
| 115 | 37.82 | gancaonin Q | C_25_H_26_O_5_ | -1 | 407.1853(+) | 351.1201[M+H-C_4_H_8_]+,295.0581[M+H-2C_4_H_8_]+,177.0176[M+H-2C_4_H_8_-C_7_H_6_O-CH_2_]+ |
| 116 | 37.85 | 3-hydroxyglabrol | C_25_H_28_O_5_ | -1 | 409.2009(+) | 409.1973,353.1324[M+H-C_4_H_8_]+,295.0659[M+H-2C_4_H_8_]+ |
| 117 | 38.73 | (E)-1-[2,4-Dihydroxy-3-(3-methyl-2-butenyl)phenyl]-3-[2,2-dimethyl-8-hydroxy-2-dione- Hydroxybenzopyran-6-yl]-2-propen-1-one | C_25_H_26_O_5_ | -1 | 407.1840(+) | 407.1893,351.1202[M+H-C_4_H_8_]+,165.0180[M+H-C_4_H_8_-C_11_H_11_O_2_-C]+ |
| 118 | 39.11 | isograbrol | C_25_H_28_O_4_ | -0.7 | 393.2060(+) | 393.2084,337.1442[M+H-C_4_H_8_]+,151.0382[M+H-C_4_H_8_-C_12_H_11_O_2_]+ |
| 119 | 39.58 | licorisoflavan A | C_27_H_34_O_5_ | 4.8 | 439.0877(+) | 327.1224[M+H-2C_4_H_8_]+, 193.0859[M+H-2C_4_H_8_-C_7_H_4_O_2_-CH_3_]+ |
| 120 | 43.86 | glycybridin D | C_25_H_26_O_4_ | 0.7 | 391.1903(+) | 149.0232[M+H-C_5_H_7_-C_11_H_11_O_2_]+ |
| 121 | 44.63 | kanzonol L | C_30_H_32_O_6_ | -1.4 | 489.2271(+) | 489.2282,433.1634[M+H-C_4_H_8_]+,377.1013[M+H-2C_4_H_8_]+,177.0186[M+H-2C_4_H_8_-C_11_H_5_O_4_]+ |

Note: “*” means comparing with reference compound

**Table S3** Peak area of 121 compounds in licorice

| No. | Putative compound | Control | SD | Salt-stressed | SD | Structure types |
| --- | --- | --- | --- | --- | --- | --- |
| 1 | glycyrrhizin | 616666.67 | 15275.25 | 721666.67 | 52519.84 | triterpenoid |
| 2 | licorice-saponin A3 | 285949.00 | 83093.64 | 162167.33 | 40668.55 | triterpenoid |
| 3 | licorice-saponin B2 | 21739.00 | 11469.15 | 119613.50 | 60192.46 | triterpenoid |
| 4 | licorice-saponin G2 | 393301.33 | 43452.78 | 218230.33 | 41040.40 | triterpenoid |
| 5 | licorice saponin H2 | 602179.67 | 73655.45 | 652952.67 | 76174.94 | triterpenoid |
| 6 | licorice-saponin J2 | 423494.00 | 32485.09 | 256032.67 | 106953.92 | triterpenoid |
| 7 | licorice-saponin K2 | 66729.00 | 14368.42 | 54065.00 | 386.91 | triterpenoid |
| 8 | uralsaponin C | 450160.67 | 36181.41 | 274619.33 | 94688.39 | triterpenoid |
| 9 | uralsaponin E | 11147.00 | 2620.54 | 276245.33 | 63095.84 | triterpenoid |
| 10 | uralsaponin F | 136767.67 | 83507.85 | 339076.00 | 16296.41 | triterpenoid |
| 11 | uralsaponin T | 4904.00 | 1630.21 | 17011.33 | 8522.27 | triterpenoid |
| 12 | uralsaponin P | 103484.67 | 8856.15 | 29747.33 | 5820.94 | triterpenoid |
| 13 | uralsaponin A | 34456.67 | 11106.71 | 38621.00 | 13475.26 | triterpenoid |
| 14 | uralsaponin B | 576190.00 | 96244.30 | 682281.00 | 74353.38 | triterpenoid |
| 15 | uralsaponin M | 142713.33 | 23616.66 | 430313.67 | 55331.08 | triterpenoid |
| 16 | uralsaponin Q | 18033.00 | 9470.96 | 29363.33 | 5623.28 | triterpenoid |
| 17 | uralsaponin R | 73333.33 | 53053.16 | 53311.67 | 50877.19 | triterpenoid |
| 18 | araboglycyrrhizin | 13031.00 | 4330.10 | 55929.67 | 15573.26 | triterpenoid |
| 19 | 3-O-β-D-glucuronopyranosyl glycyrrhetinic acid | 383092.00 | 254479.68 | 741750.00 | 33762.93 | triterpenoid |
| 20 | 22β-acetoxyl-glyrrhaldehyde | 5020.23 | 1670.25 | 118279.50 | 9454.72 | triterpenoid |
| 21 | glycyrrhetinic acid | 285357.67 | 15038.84 | 316038.33 | 28287.99 | triterpenoid |
| 22 | glabric acid | 328389.67 | 117350.76 | 327554.50 | 5328.05 | triterpenoid |
| 23 | 7',4-dihydroxyflavone | 159642.67 | 25694.26 | 121847.33 | 61194.48 | flavone |
| 24 | licoflavone C | 154170.33 | 4344.93 | 178447.00 | 36040.34 | flavone |
| 25 | gancaonin Q | 367688.00 | 179204.11 | 423549.67 | 44948.16 | flavone |
| 26 | apigenin | 752032.50 | 14267.29 | 505169.33 | 172281.99 | flavone |
| 27 | glycyroside | 125542.00 | 23082.69 | 83429.33 | 31693.23 | isoflavone glycosides |
| 28 | ononin | 503801.67 | 120372.23 | 563832.00 | 113621.20 | isoflavone glycosides |
| 29 | schaftoside | 25031.33 | 27.14 | 22298.00 | 7029.68 | flavone glycosides |
| 30 | sophoraflavone B | 38047.33 | 8701.25 | 57291.33 | 26521.16 | flavone glycosides |
| 31 | 7,4'-Dihydroxyflavone-7-O-glucoside | 0.00 | 0.00 | 3179.33 | 794.02 | flavone glycosides |
| 32 | licorice glycoside D2 | 27283.00 | 8652.22 | 20734.00 | 6308.29 | flavone glycosides |
| 33 | neoliquiritin | 110160.00 | 17181.94 | 489438.00 | 92145.42 | flavone glycosides |
| 34 | naringin | 83288.00 | 22630.86 | 96681.67 | 43449.59 | flavone glycosides |
| 35 | kaempferin | 23019.31 | 5754.75 | 23268.33 | 6780.69 | flavone glycosides |
| 36 | quercitrin | 19990.67 | 6936.29 | 17631.50 | 7500.28 | flavone glycosides |
| 37 | isoononin | 468687.67 | 220922.86 | 543024.67 | 123650.40 | flavone glycosides |
| 38 | nicotiflorin | 43248.00 | 3249.33 | 76508.67 | 10911.54 | flavone glycosides |
| 39 | uralenol | 168003.00 | 56010.00 | 74749.33 | 22561.02 | flavonol |
| 40 | topazolin | 228428.67 | 41940.46 | 143052.67 | 61629.83 | flavonol |
| 41 | angustone A | 251000.00 | 95712.33 | 332098.67 | 24367.44 | isoflavone |
| 42 | glicoricone | 209605.33 | 98921.81 | 186839.67 | 32321.03 | isoflavone |
| 43 | licoisoflavone A | 362135.33 | 108243.55 | 1280763.00 | 322993.43 | isoflavone |
| 44 | 2'-hydroxyisolupalbigenin | 656942.33 | 326762.56 | 459564.33 | 215997.04 | isoflavone |
| 45 | genistein | 658855.67 | 136048.91 | 557464.33 | 288186.41 | isoflavone |
| 46 | formononetin | 716221.00 | 298128.57 | 460830.00 | 50888.30 | isoflavone |
| 47 | abiochanin A | 77719.00 | 24591.83 | 39313.00 | 19418.57 | isoflavone |
| 48 | glicophenone | 46565.67 | 15469.72 | 34901.67 | 10734.03 | isoflavone |
| 49 | isoglabrone | 331625.33 | 106244.55 | 314404.67 | 127583.33 | isoflavone |
| 50 | eurycarpin A | 130802.33 | 40990.56 | 152945.67 | 21497.24 | isoflavone |
| 51 | glyurallin B | 147799.00 | 19350.92 | 522474.00 | 196432.82 | isoflavone |
| 52 | glycyrrhisoflavone | 262988.00 | 14699.56 | 19315.33 | 903.00 | isoflavone |
| 53 | 7,3',4'-trihydroxyisoflavone | 660376.00 | 94894.64 | 609796.00 | 198129.63 | isoflavone |
| 54 | calycosin | 698855.67 | 101225.62 | 530863.33 | 272243.07 | isoflavone |
| 55 | kanzonol L | 34481.00 | 16383.73 | 8757.00 | 3799.99 | isoflavone |
| 56 | 8-methylretusin | 58891.67 | 30739.95 | 50316.33 | 21786.18 | isoflavone |
| 57 | 3'-isoprenylgenistein | 75384.67 | 23033.54 | 151313.67 | 9946.31 | isoflavone |
| 58 | orobol | 164545.00 | 46974.25 | 70631.67 | 1426.52 | isoflavone |
| 59 | 7-methyl-luteone | 222346.00 | 66265.08 | 143014.00 | 68869.51 | isoflavone |
| 60 | cycloglycyrrhisoflavone | 288337.33 | 83682.88 | 100271.33 | 35158.98 | isoflavone |
| 61 | echinatin | 567754.33 | 84669.99 | 523144.33 | 166702.61 | chalcone |
| 62 | isoliquiritigenin | 261117.00 | 88671.17 | 205117.67 | 53886.64 | chalcone |
| 63 | homobutein | 41685.33 | 18228.42 | 28899.33 | 9344.60 | chalcone |
| 64 | licochalcone A | 227600.33 | 33067.80 | 159692.67 | 54031.10 | chalcone |
| 65 | 5-(1,1-dimethylallyl)-3,4,4g-trihydroxy-2-methoxychalcone | 1264419.67 | 323202.84 | 1172950.33 | 364655.31 | chalcone |
| 66 | licoagrochalcone C | 270857.00 | 20297.65 | 251466.67 | 50093.55 | chalcone |
| 67 | 4,2',3'-trihydroxy-4'-methoxy-chalcone | 72559.33 | 7210.49 | 24267.50 | 23021.28 | chalcone |
| 68 | corylifol B | 43068.00 | 15470.73 | 16954.67 | 3237.77 | chalcone |
| 69 | 3,4,3',4'-Tetrahydroxychalcone | 91982.00 | 25419.73 | 86351.33 | 20290.36 | chalcone |
| 70 | glypallichalone | 77719.00 | 24591.83 | 42061.00 | 15532.31 | chalcone |
| 71 | (E)-1-[2,4-Dihydroxy-3-(3-methyl-2-butenyl)phenyl]-3-[2,2-dimethyl-8-hydroxy-2-dioneHydroxybenzopyran-6-yl]-2-propen-1-one | 10288.67 | 1116.33 | 19775.33 | 8731.54 | chalcone |
| 72 | licoagrochalcone | 44804.00 | 10295.40 | 7563.00 | 1892.23 | chalcone |
| 73 | tetrahydroxy-methoxychalcone | 9839.67 | 4537.55 | 21797.33 | 7148.93 | chalcone |
| 74 | glycybridin A | 218228.00 | 42685.59 | 63507.67 | 22453.18 | chalcone |
| 75 | [6'',6''-dimethylpyrano(2'',3'':4,5)]-3'-γ,γ-dimethylallyl-2',3,4'-trihydroxychalcone | 101150.33 | 27410.61 | 406447.67 | 26892.41 | chalcone |
| 76 | isoliquiritigenin-4,4'-diglucoside | 86394.33 | 18590.40 | 97346.67 | 43558.12 | chalcone glycosides |
| 77 | isoliquiritin apioside | 169377.33 | 9656.04 | 431636.33 | 63526.72 | chalcone glycosides |
| 78 | isoliquiritin | 143630.00 | 68558.78 | 257735.33 | 85032.18 | chalcone glycosides |
| 79 | licorice-glycoside B | 123508.33 | 45215.65 | 91594.00 | 34767.03 | chalcone glycosides |
| 80 | licuraside | 360234.33 | 70001.47 | 468379.00 | 106338.35 | chalcone glycosides |
| 81 | paratocarpin L | 42082.00 | 18236.32 | 42943.67 | 21377.97 | flavanone |
| 82 | isograbrol | 14219.00 | 5335.06 | 22790.00 | 8807.72 | flavanone |
| 83 | licoflavanone | 194894.67 | 57717.88 | 63507.67 | 22453.18 | flavanone |
| 84 | uralenin | 83064.00 | 43854.32 | 4887.00 | 178.45 | flavanone |
| 85 | cyclolicoflavanone | 195381.67 | 61675.62 | 63507.67 | 22453.18 | flavanone |
| 86 | naringenin | 537516.00 | 98668.45 | 228899.67 | 113778.42 | flavanone |
| 87 | liquiritigenin | 135450.67 | 67940.27 | 125191.67 | 5493.63 | flavanone |
| 88 | glyasperin B | 348384.00 | 173558.91 | 117253.67 | 29673.76 | flavanone |
| 89 | (2R,3R)-3,4',7-trihydroxy-3'-prenylflavane | 206456.00 | 55030.72 | 50740.50 | 5503.41 | flavanone |
| 90 | 3-hydroxyglabrol | 37839.33 | 20057.73 | 49723.00 | 12274.45 | flavanone |
| 91 | glucoliguiritin apioside | 51968.00 | 24868.02 | 77634.33 | 29683.46 | flavanone glycosides |
| 92 | liquiritin apioside | 37733.33 | 7327.30 | 45570.00 | 18268.10 | flavanone glycosides |
| 93 | liquiritin | 75717.67 | 22868.95 | 54957.00 | 23972.48 | flavanone glycosides |
| 94 | liquiritigenin-7-O-β-D-(3-O-acetyl)furanose-4'-O-β-D-glucopyranoside | 11532.67 | 6029.06 | 25930.00 | 9911.48 | flavanone glycosides |
| 95 | choerospondin | 6946.50 | 1772.72 | 45352.00 | 15107.22 | flavanone glycosides |
| 96 | licorice-glycoside C1 | 16588.33 | 5661.26 | 15101.50 | 5653.32 | flavanone glycosides |
| 97 | licoricidin | 833214.33 | 330201.48 | 1104645.67 | 56190.34 | isoflavan |
| 98 | glyasperin D | 350303.00 | 100165.42 | 316253.00 | 124062.64 | isoflavan |
| 99 | glyasperin C | 421057.67 | 177358.67 | 466673.00 | 251966.10 | isoflavan |
| 100 | gancaonin Z | 33773.67 | 21975.72 | 12047.00 | 1473.33 | isoflavan |
| 101 | licorisoflavan A | 19714.33 | 3375.95 | 858877.00 | 47315.79 | isoflavan |
| 102 | licorisoflavan B | 457190.67 | 59179.13 | 460413.33 | 203305.73 | isoflavan |
| 103 | gancaonol C | 139959.00 | 50487.18 | 207189.67 | 9043.76 | isoflavan |
| 104 | dehydroglyasperin D | 102665.00 | 21425.65 | 137683.33 | 49735.36 | isoflavan |
| 105 | isovestitol | 113865.67 | 48682.43 | 83847.00 | 3542.22 | isoflavan |
| 106 | glycycoumarin | 80187.00 | 18982.40 | 51090.33 | 2080.99 | coumarin |
| 107 | trifolirhizin | 21599.33 | 7414.19 | 59892.00 | 15753.08 | coumarin |
| 108 | glyurallius A | 21000.00 | 5334.00 | 8684.00 | 3203.19 | coumarin |
| 109 | licoagroside | 49039.67 | 14892.54 | 34177.33 | 5761.92 | coumarin |
| 110 | hedysarimcoumestan E | 43813.33 | 15148.36 | 196389.67 | 61703.75 | coumarin |
| 111 | 7,2',4'-trihydroxy-8,3'-diisoprenyl-3-phenyl coumarin | 103107.67 | 38742.56 | 441411.33 | 21254.26 | coumarin |
| 112 | glycyuralin E | 141534.33 | 29750.09 | 275759.67 | 119118.85 | coumarin |
| 113 | licocoumarone | 75705.67 | 11419.70 | 30500.00 | 5232.86 | coumarin |
| 114 | licopyranocoumarin | 94672.00 | 27223.31 | 332692.67 | 135720.19 | coumarin |
| 115 | licoagrocarpin | 228065.33 | 32886.21 | 217734.33 | 2606.31 | coumarin |
| 116 | neoglycyrol | 183540.33 | 39076.27 | 125347.50 | 3369.36 | coumarin |
| 117 | glycybridin D | 17979.00 | 7116.68 | 21732.33 | 2062.10 | coumarin |
| 118 | glycybridin H | 493010.67 | 110021.08 | 605464.00 | 58506.67 | coumarin |
| 119 | glyurallin A | 81836.00 | 16021.06 | 241028.00 | 20072.51 | coumarin |
| 120 | 2'-methoxyisoliquiritigenin | 481673.67 | 422255.32 | 583195.00 | 185732.22 | others |
| 121 | cyclolicocoumarone | 18425.33 | 3177.22 | 4688.33 | 539.73 | others |
